# The tendon interfascicular basement membrane provides a vascular niche for CD146+ cell subpopulations

**DOI:** 10.3389/fcell.2022.1094124

**Published:** 2023-01-09

**Authors:** Neil Marr, Danae E. Zamboulis, Dirk Werling, Alessandro A. Felder, Jayesh Dudhia, Andrew A. Pitsillides, Chavaunne T. Thorpe

**Affiliations:** ^1^ Comparative Biomedical Sciences, Royal Veterinary College, London, United Kingdom; ^2^ Pathobiology and Population Sciences, Centre for Vaccinology and Regenerative Medicine, Royal Veterinary College, Hatfield, United Kingdom; ^3^ Research Software Development Group, Advanced Research Computing, University College London, London, United Kingdom; ^4^ Clinical Sciences and Services, Royal Veterinary College, Hatfield, United Kingdom

**Keywords:** tendon, interfascicular matrix, tendon progenitors, CD146, basement membrane

## Abstract

**Introduction:** The interfascicular matrix (IFM; also known as the endotenon) is critical to the mechanical adaptations and response to load in energy-storing tendons, such as the human Achilles and equine superficial digital flexor tendon (SDFT). We hypothesized that the IFM is a tendon progenitor cell niche housing an exclusive cell subpopulation.

**Methods:** Immunolabelling of equine superficial digital flexor tendon was used to identify the interfascicular matrix niche, localising expression patterns of CD31 (endothelial cells), Desmin (smooth muscle cells and pericytes), CD146 (interfascicular matrix cells) and LAMA4 (interfascicular matrix basement membrane marker). Magnetic-activated cell sorting was employed to isolate and compare in vitro properties of CD146+ and CD146− subpopulations.

**Results:** Labelling for CD146 using standard histological and 3D imaging of large intact 3D segments revealed an exclusive interfascicular cell subpopulation that resides in proximity to a basal lamina which forms extensive, interconnected vascular networks. Isolated CD146+ cells exhibited limited mineralisation (osteogenesis) and lipid production (adipogenesis).

**Discussion:** This study demonstrates that the interfascicular matrix is a unique tendon cell niche, containing a vascular-rich network of basement membrane, CD31+ endothelial cells, Desmin+ mural cells, and CD146+ cell populations that are likely essential to tendon structure and/or function. Contrary to our hypothesis, interfascicular CD146+ subpopulations did not exhibit stem cell-like phenotypes. Instead, our results indicate CD146 as a pan-vascular marker within the tendon interfascicular matrix. Together with previous work demonstrating that endogenous tendon CD146+ cells migrate to sites of injury, our data suggest that their mobilisation to promote intrinsic repair involves changes in their relationships with local interfascicular matrix vascular and basement membrane constituents.

## 1 Introduction

Tendons are fundamental components of the musculoskeletal system, acting as connections between muscle and bone. The predominant function of tendon is to transfer the forces exerted by skeletal muscle contractions to bone, positioning the limb for locomotion (Alexander, 1991; Benjamin et al., 2008). However, specialised energy-storing tendons, such as the equine superficial digital flexor tendon (SDFT) and human Achilles tendon, enhance the functional adaptation of tendon by lowering the energetic cost of locomotion through their mechanical properties, such as greater extensibility, elasticity and fatigue resistance (Biewener, 1998; Alexander, 2002; Thorpe et al., 2015). Much like skeletal muscle, these specialised mechanical properties of energy-storing tendons are provided by their hierarchical structure of subunits predominantly composed of type I collagen, forming fascicles and fascicle bundles. Both fascicles and fascicle bundles are surrounded and bound by a non-collagenous interfascicular matrix (IFM, also known as the endotenon) which governs the high-strain behaviour of energy-storing SDFT by facilitating sliding between fascicles (Kannus, 2000; Thorpe and Screen, 2016; Handsfield et al., 2016). While the mechanical role of IFM in the function of energy storing tendons is well defined, less is known regarding its biological role in developing, adult and ageing tendon, particularly regarding the identity and function of IFM localised cell populations and their niche, defined as the anatomical microenvironment in which specific cell populations reside.

Histological analyses of tendon have revealed regional morphological differences in cell populations, with rounder cells within the IFM, present in greater number compared to those within the fascicles which are highly aligned with the long axis of the tendon (Thorpe and Screen, 2016; Thorpe et al., 2016). In addition, seminal studies have alluded to an endogenous tendon stem/progenitor cell (TSPC) population and niche, both of which remain largely undefined but have been speculated to reside within the IFM (Bi et al., 2007; Richardson et al., 2007; Godwin et al., 2012). In other tissues, the stem cell niche is maintained by mechanically unique microenvironments, similar to the high shear environment within the IFM, which may therefore be the location of the tendon stem/progenitor cell niche (Evans et al., 2013; Ivanovska et al., 2015; Smith et al., 2018).

Tendon development is driven by stem/progenitor cell populations which express Mohawk homeobox (MKX) and Scleraxis (SCX) transcription factors (Schweitzer et al., 2001; Anderson et al., 2006; Kimura et al., 2011); unfortunately, their intracellular localisation impedes cell sorting techniques required for *in vitro* study of stem/progenitor cell populations. In adult tissues, cell surface markers, such as CD44 and CD90 (THY1), are members of a number of canonical marker panels routinely used in the characterisation and isolation of specific stromal/stem cell populations (Horwitz et al., 2005; Morath et al., 2016). Recent studies have also reported resident CD146 populations in tendon (Yin et al., 2016; Gumucio et al., 2020). CD146 or melanoma adhesion molecule (MCAM; MUC18; Gicerin; OMIM:155735) is a transmembrane glycoprotein belonging to the IgG superfamily of cell adhesion molecules (Shih, 1999). Originally characterised as a marker of tumour progression and metastasis, CD146 has since been reported as a marker of endothelial cell lineages, both haematopoietic and mesenchymal stem cell lineages, as well as synovial fibroblasts and periosteal cells (Johnson et al., 1993; Sers et al., 1993; Schlagbauer-Wadl et al., 1999; Schrage et al., 2008; Kaltz et al., 2010; Russell et al., 2010; Tormin et al., 2011). Our laboratory has recently reported that these CD146 subpopulations are present within the IFM of the rat Achilles and recruited to injury sites from their IFM niche *via* the CD146 ligand Laminin α4 (LAMA4) (Marr et al., 2021). However, to the authors’ knowledge, no studies have attempted to comprehensively characterise CD146 tendon cells and their *in vivo* cell niche composition.

In this study, we tested the hypothesis that the IFM is a tendon progenitor cell niche housing an exclusive cell subpopulation. We report novel markers of interfascicular cells and basement membrane, and identify CD146 as an optimal marker for use in IFM cell sorting procedures. We also demonstrate that the lineage potential and clonogenicity of interfascicular CD146 cells is limited, which may be indicative of a differentiated vascular population rather than resident tendon stem/progenitor cells.

## 2 Materials and methods

### 2.1 Ethical statement

The collection of animal tissues was approved by the Royal Veterinary College Ethics and Welfare Committee (URN-2016-1627b). All tissues were sourced from horses euthanised for reasons unrelated to this study, and other than tendon injury at a commercial equine abattoir.

### 2.2 Tissue acquisition

Superficial digital flexor tendons (SDFT) were harvested from forelimbs taken from young, skeletally mature horses (age = 3–8 years, *n* = 5, exercise history unknown). Prior to isolation, the forelimbs were clipped to remove hair and the skin sterilised by several applications of 4% chlorhexidine (HiBiScrub®; Mölnlycke Health Care). Portions of mid-metacarpal SDFT (6–10 cm) were dissected free of the limb and stored immediately in standard growth medium consisting of pyruvate and low glucose Dulbecco’s modified eagle medium (DMEM) supplemented with 1% (v/v) penicillin/streptomycin and 10% (v/v) qualified, heat-inactivated foetal bovine serum (FBS) until tissue processing (all from Gibco™). Excised tendons presenting with previously reported definitions of macroscopic evidence of injury were excluded from all experiments (Webbon, 1977; Dakin et al., 2012), and all tendons had a normal histological appearance. Dissections and subsequent cell processing were completed within 24 h of euthanasia.

### 2.3 Cryosectioning

SDFT frozen sections were prepared as previously described (Godinho et al., 2017). Tissues were briefly washed in Dulbecco’s phosphate-buffered saline (calcium and magnesium free, embedded with optimal cutting temperature compound (OCT; Cell Path, Newtown, United Kingdom) embedding matrix and snap-frozen in pre-cooled hexane on dry ice. Serial longitudinal (6–20 µm thickness) and transverse (30 µm thickness) sections were prepared using a cryostat microtome (OTF5000, Bright Instruments) equipped with MX35 Premier Disposable Low-Profile Microtome Blades (3052835, Fisher Scientific). Tissue sections were mounted on SuperFrost™ Plus Slides (10149870, Fisher Scientific), air-dried at room temperature (RT) for a maximum of 2 h and stored at −80 °C.

### 2.4 Periodic acid-Schiff staining

Periodic acid-Schiff (PAS) staining was used to detect mucins and basement membrane proteins. Staining was performed using an Alcian Blue (pH 2.5)/PAS staining kit according to manufacturer guidelines (Atomic Scientific). SDFT cryosections (20 µm) were thawed and fixed with 4% PFA/10% NBF for 10 min at RT. Slides were rinsed thoroughly with distilled water, stained with 1% Alcian blue in 3% acetic acid (pH 2.5) for 10 min, and washed thoroughly in distilled water. Slides were treated with 1% periodic acid solution for 10 min at RT, washed with distilled water, then treated with Schiff reagent (Feulgen) for 10 min at RT. Sections were then washed under running tap water until sections presented a magenta colour macroscopically. Sections were then counter-stained with haematoxylin, dehydrated and cleared using an automated slide stainer (Varistain^™^ Gemini ES), and mounted with glass coverslips using DPX mountant. Slides were cured at RT overnight and imaged using brightfield microscopy (DM4000B upright microscope) in Leica Application Suite software version 2.6 (Leica Microsystems).

### 2.5 Network-based predictions of CD146 interactions

Proteins of interest for immunolabelling were selected based on their expression by tendon progenitor cell subpopulations in previous reports (Yin et al., 2016) and their predicted interactions in *Equus caballus* (NCBI taxid: 9796) using STRING (version 10.5) network-based predictions for CD146 and LAMA4 (Szklarczyk et al., 2017; Gumucio et al., 2020).

### 2.6 Immunolabelling

SDFT cryosections were thawed and fixed with acetone (pre-cooled at −20°C) for 10 min, washed three times for 5 min at RT with tris-buffered saline (TBS), incubated in “blocking” buffer (TBS supplemented with 1% (w/v) bovine serum albumin (Scientific Laboratory Supplies), 5% (v/v) goat serum (Sigma), and 5% (v/v) horse serum (Sigma) for 2 h. Horse serum was used to saturate Fc receptors on the surface of cells within the tissue. Sections were incubated with primary antibodies overnight at 4°C (details regarding primary and secondary antibodies are provided in Supplementary Table S1). For negative controls, sections were treated with blocking buffer only. For isotype controls, sections were treated with mouse and rabbit IgG isotype-matched controls diluted in blocking buffer at identical concentration to primary antibodies used. For fluorescent detection (10 µm sections), secondary antibodies diluted in blocking buffer were applied to sections and incubated for 1 h at RT under dark conditions. Sections were washed three times with TBS for 5 min, and mounted with glass coverslips using ProLong^™^ Diamond antifade mountant with 4′,6-diamidino-2-phenylindole (DAPI) as a nuclei counterstain. Slides were cured for 24 h at RT under dark conditions, prior to imaging. Negative and isotype matched control images for fluorescent labelling are provided in Supplementary Figure S1. Immunohistochemical labelling (6 µm sections) was performed in a similar manner to fluorescent detection, using an EnVision®^+^ Dual Link System-HRP DAB^+^ system (Dako), with the inclusion of an of EnVision dual endogenous enzyme block for 15 min at RT under dark conditions prior to treatment with blocking buffer, and wash steps were performed using .05% (v/v) TBS-TWEEN20. For immunohistochemical detection, sections were incubated in EnVision peroxidase labelled polymer (conjugated to goat anti-mouse and goat anti-rabbit immunoglobulins) for 30 min at RT. Sections were then washed three times and incubated with EnVision DAB^+^ substrate buffer-3,3′-diaminobenzidine (DAB) chromogen solution for 3 min, rinsed three times with deionised water (diH_2_O), counter-stained using haematoxylin according to Delafield, dehydrated and cleared using standard procedures on a Varistain^™^ Gemini ES automated slide stainer, then finally mounted with glass coverslips using DPX mountant. Slides were cured at RT overnight and imaged using brightfield microscopy (DM4000B upright microscope) in Leica Application Suite software version 2.6 (Leica Microsystems). Regions clearly showing IFM vascular morphology and positively labelled structures were chosen to demonstrate protein localisation. Negative control images for immunohistochemistry are provided in Supplementary Figure S2.

### 2.7 Fluorescent labelling analyses

To distinguish between regions of IFM and fascicular matrix (FM), boundaries between both phases were determined by light refraction in phase contrast images, as well as gross identification by nuclei number and cell morphology (Supplementary Figure S3). For quantification, all settings remained constant between samples including exposure, pixel size, z-step size, and laser settings with all images taken in one single session. For each sample, two distinct areas were imaged in two separate serial tissue sections (2 × sections per horse donor, *n* = 5). Confocal images are presented as maximum intensity projections from z-stacks containing image slices at a resolution of 512 × 512 × 40 pixels (227.9 × 227.9 × 13.09 µm; .34 µm z-step size) to fully capture tissue depths. Image processing and analysis was performed using Fiji/ImageJ software (Schindelin et al., 2012). For IFM measurements, an area fraction (%) of positively stained pixels were recorded in 8-bit binary images (black = negative, white = positive) to measure expression of markers of interest. To generate binary images for each marker, a background correction was performed to remove noise, followed by a median filter and threshold (Triangle for CD146/MKX = 555 nm, Huang for CD44/CD90 = 633 nm). The lookup table (LUT) of colour channels within images was changed for visualisation purposes.

### 2.8 3D immunolabelling

3D immunolabelling of SDFT segments was performed as previously described (Marr et al., 2020). All steps were performed with orbital agitation. SDFT segments (5 mm × 5 mm  ×  2 mm, *n* = 2) were washed twice for 12 h with TBS at RT, and permeabilised sequentially in 50% (v/v) methanol:TBS, 80% (v/v) methanol:diH_2_O, and 100% methanol for 2 h at 4°C. Samples were washed sequentially for 40 min at 4°C with 20% (v/v) DMSO:methanol, 80% (v/v) methanol:diH_2_O, 50% (v/v) methanol:TBS, TBS, and TBS supplemented with .2% (v/v) Triton X-100. Prior to blocking, samples were incubated with a pre-blocking penetration buffer containing .2% TBS-TX100, .3 M glycine, and 20% DMSO for 6 h at 37°C. Equine SDFT segments were blocked for 80 h at 37°C in .2% TBS-TX100 supplemented with 6% (v/v) goat and 6% (v/v) donkey serum and 10% (v/v) DMSO. Primary antibody incubations for CD146 (1:100) were performed at 37°C for 80 h in wash buffer (TBS supplemented with .2% (v/v) TWEEN20), 3% (v/v) goat serum, 3% (v/v) donkey serum, and 5% (v/v) DMSO. Segments were washed 3 × 2 h with wash buffer, incubated with secondary antibodies (1:250, goat anti-rabbit Alexa Fluor® 594) for 36 h at 37°C, washed 5 × 5 min with wash buffer, and counterstained overnight with DAPI (1:2000) diluted in wash buffer. Segments were dehydrated with increasing concentration of methanol, and tissue cleared with immersion in Visikol® HISTO™-1 for 36 h, followed by immersion in HISTO™-2 for at least 36 h at RT. Samples were stored in HISTO™-2 at 4°C prior to confocal imaging. Confocal imaging of regions (approx. 1 mm × 1 mm  × .2 mm) within each sample was performed using a Leica TCS SP8 laser scanning confocal microscope with a motorised stage. Images were captured using lasers emitting light at 405 (blue channel; DAPI) and 561 (red channel; Alexa Fluor 594) nm with laser power <10% and scanning speed = 600 Hz with a HC PL FLUOTAR 10x/.32 dry objective lens, resolution = 1,024 × 1,024 px, pinhole size = 1 Airy unit, frame average = 1, line average = 8, and electronic zoom = .75. 3D renderings were captured in Leica LAS X software (version 3.5.5) within the 3D module.

### 2.9 Primary tendon cell culture

SDFTs collected under sterile conditions were placed in Petri dishes containing Gibco™ Dulbecco’s PBS (without phenol red, calcium and magnesium) supplemented with 1% (v/v) antibiotic-antimycotic solution. Surrounding peritenon was removed to isolate the tendon core (6 g), which was diced into approximately 4 mm^3^ pieces, rinsed with DPBS, and digested with 1 mg/mL pronase E (39052, VWR) per 1 g tissue for 6–8 h at 37°C and 5% CO_2_ under constant agitation. Following pronase digestion, tissue was digested for a further 24 h with .5 mg/ml collagenase type IV (CLS-4, Lorne Laboratories) and 1 mg/mL dispase II (17105041, Invitrogen) at 37°C and 5% CO_2_ with constant agitation (Garvican et al., 2017).

### 2.10 Magnet-activated cell sorting (MACS) of CD146 cells

Previous studies have shown that >50% expression of cell membrane proteins can be restored post-digestion by 24 h *in vitro* culture (Autengruber et al., 2012). Hence, to enhance antigen recovery, freshly digested tendon-derived cells (TDCs) were cultured overnight to maximise CD146 cell isolations. Following this recovery phase, adherent cells were dissociated at 37°C for 10 min using Accutase® solution according to manufacturer’s guidelines. Cells remaining in suspension (i.e. non-adherent populations) were also collected alongside dissociated cells (adherent populations). Cell isolates were washed by resuspension in fresh growth medium and centrifuged at 300 × g for 10–20 min depending on pellet formation. Cell pellets (passage 1; p1) were resuspended in growth medium and separated into single-cell suspensions (SCSs) by passing through a 70 μm cell strainer. SCSs were resuspended in freshly prepared, ice-cold MACS buffer containing sterile-filtered FACSFlow™ (342003, BD Biosciences) supplemented with 1% (w/v) BSA. SCSs were centrifuged for 10 min at 300 × g, resuspended in MACS buffer, and both cell viability and numbers determined by trypan blue (T8154, Sigma-Aldrich) and a haemocytometer. Suspensions with <90% viability were discarded. SCSs were incubated with anti-CD146 antibodies (ab75769, Abcam, Cambridge, United Kingdom) at a concentration of 1 μg/mL for 30 min at 4°C on ice. Following primary antibody incubation, SCSs were washed three times by centrifugation at 300 × g, resuspended in MACS buffer, and incubated with anti-rabbit IgG micro-beads (130-048-602, Miltenyi biotec) diluted in MACS buffer for 15 min at 4°C. SCSs were washed three times by centrifugation at 300 × g and resuspended in MACS buffer. MidiMACS™ LS columns (130-042-401, Miltenyi biotec) were mounted to a MidiMACS™ Separator and multistand (130-042-301, Miltenyi biotec) and washed with MACS buffer according to manufacturer guidelines. MACS-ready SCSs were passed through MidiMACS™ columns and washed with MACS buffer twice. All wash elutions containing negatively selected cells (i.e. CD146^-^ TDCs) were collected on ice until processing of sub-cultures. Following negative cell depletion, CD146^+^ cells were collected by removing the MACS column from the MACS magnet and eluting the column with MACS buffer and a plunger. All sub-cultures were maintained until a maximum of three passages (p3) to limit phenotypic drift. For downstream assays, cells were dissociated using Accutase® solution (A6964, Sigma-Aldrich). *n* = 9 (biological replicates).

### 2.11 Flow cytometry

For direct flow cytometry, .1–.2 × 10^6^ cells were resuspended in DPBS. For CD146^+^ cells, lower concentrations were used according to yields following MACS isolation. All tubes were stored on ice immediately prior to and during flow cytometry. Cell suspensions (50 µl) were incubated with a phycoerythrin (PE)-conjugated variant of the EPR3208 anti-CD146 antibody (1:100, ab209298, Abcam, Cambridge, United Kingdom) on ice for 30 min, washed with DPBS and spun at 400 × g. Supernatant was removed, and pellets resuspended in 500 µl DPBS for immediate flow cytometry analyses.

All flow cytometry acquisition was performed using an air-cooled 3-laser BD FACSCanto II™ flow cytometer (BD Biosciences) equipped with BD FACSDiva (version 8.0.1, BD Biosciences). Acquisition equipment and software were calibrated daily or immediately prior to acquisition using BD FACSDiva™ CS&T Research Beads (BD Biosciences). Data analyses was performed in FlowJo software (version 10, FlowJo LLC). Unstained controls (fluorescence minus one control) were used to gate and discriminate positively and negatively labelled populations (see Supplementaty Figure S4). The percentage of positive cells gated in unstained samples (i.e., autofluorescent cells) was subtracted from stained samples (i.e. experimental cells) to give an overall percentage of immunoreactivity. All experiments recorded a minimum of 10,000 total events (i.e. cells). *n* = 2 (biological replicates) per cell fraction.

### 2.12 Immunocytochemistry

For detection of CD146 in unsorted TDCs, .1–.2 × 10^6^ cells were seeded on sterile 16 mm borosilicate glass circle coverslips coated with poly-L-lysine solution (.01%, sterile-filtered, P4832, Sigma-Aldrich) until 70%–80% confluence. To detect CD146 within MACS-enriched CD146^+^ cells, immunocytochemistry was performed directly on cells (.1–.2 × 10^6^ seeding density) in non-coated culture vessels at 70%–80% confluence. Cells were washed 3 times with DPBS, fixed with pre-chilled (−20°C) acetone:methanol (1:1) for 20 min on ice, then washed three times with DPBS. Cells were blocked for 1 h with blocking buffer as described above. Cells were incubated overnight with primary antibodies overnight at 4°C as described above, washed three times with DPBS, incubated for 1 h with secondary antibodies (1:500, goat anti-rabbit Alexa Fluor® 488 and goat anti-mouse Alexa Fluor® 594).

For direct CD146 labelling in MACS-sorted populations, cells were incubated overnight at 4 °C with phycoerythrin (PE)-conjugated anti-CD146 antibodies (1:100, ab209298, Abcam, Cambridge, United Kingdom). Cells were washed three times with DPBS, labelled with DAPI (1 μg/mL) for 2 min, washed three times with DPBS and mounted using Prolong™ Diamond, cured at RT under dark conditions for 24 h before storing at 4°C until imaging. Fluorescent imaging of TDCs was performed using a Leica SP5 (40 × HCX PL FLUOTAR PH2 NA = .75 objective). For CD146^+^ cells, imaging was performed on a DMIRB inverted microscope (Leica Microsystems, Wetzlar, Germany; 40 × N PLAN L corr PH2 NA = .55 objective). *n* = 3 (biological replicates).

### 2.13 Clonogenic assay

Bone marrow-derived mesenchymal stromal cells (MSCs) isolated as described previously were kindly provided by Dr Giulia Sivelli (Godwin et al., 2012). MSCs, unsorted TDCs, sorted CD146^−^ cells and CD146^+^ cells were seeded in 6-well plates at a density of 100 cells cm^−3^ (approx. 900 cells) and cultured for 7 d. At termination of cultures, cells were washed 3× with DPBS, fixed with 2.5% glutaraldehyde for 10 min, then washed 3× DBPS (all steps at RT). Cells were stained with .1% (v/v) crystal violet for 30 min at RT. Cells were washed 3x with DBPS and left to air dry at RT. Images were acquired using a flat-bed scanner (Epson Perfection 4990, Epson) at a resolution of 800 dpi. *n* = 3 for each cell type (biological replicates). *n* = 2–3 wells for each condition (technical replicates).

### 2.14 Adipogenesis assay

MSCs, unsorted TDCs, sorted CD146^−^ cells and CD146^+^ cells were seeded into 12-well plates at a density of .4 × 10^5^ cells per well and cultured for 48 h until adherence in standard growth medium. To induce adipogenesis, standard growth media was removed, and cells were cultured with StemPro® Adipogenesis differentiation media for a further 14 d. Cells were fed induction media every 72 h. Upon termination of culture, monolayers were washed once with DPBS before fixation with 4% PFA/10% NBF for 30 min at RT. To assess intracellular lipid vesicles produced by adipogenic conditions, cells were stained with Oil Red O. Fixed monolayers were rinsed once with distilled water then washed with 60% isopropanol for 5 min at RT. Monolayers were stained for 15 min at RT with a 3:2 working solution of 3-parts .3% (w/v) Oil Red O diluted in isopropanol and 1-part distilled water. Cells were washed repeatedly with distilled water until rinsed clear of precipitating Oil Red O, then counterstained with Harris haematoxylin for 1 min at RT. Imaging was performed on an Axiovert 135TV inverted microscope (Zeiss) using Image Pro Insight version 9.1.4 (Media Cybernetics). *n* = 3 for each cell type (biological replicates). *n* = 2-3 wells for each condition (technical replicates).

### 2.15 Osteogenesis assay

Following dissociation, MSCs, unsorted TDCs, sorted CD146^+^ and CD146^-^ cells were seeded into 12-well plates a density of .1 × 10^6^ cells per well with osteogenic media containing 2 mM sodium phosphate dibasic (DiP) or standard growth medium as a control with each condition supplemented with 50 μg/ml ascorbic acid to promote collagen synthesis (Barnes, 1975; Patel et al., 2019). DiP (free phosphate donor) is essential for bone/mineralised extracellular matrix metabolism during osteogenesis (Robey and Termine, 1985). Monolayers were fed with fresh half-media changes corresponding to each condition every 72 h. Cell cultures were terminated after 21 days to assess mineralisation with Alizarin Red S staining (Taylor et al., 2014). Monolayers were rinsed once with DPBS then fixed for 10 min at RT with 2.5% (v/v) glutaraldehyde. Fixed cells were rinsed once with DBPS then three times with 70% ethanol and air-dried at RT overnight. Dried monolayers were subsequently stained with 1% (w/v) Alizarin Red S in diH_2_O for 5 min at RT, then washed three times with 50% ethanol and left to air-dry overnight. Imaging was performed as described above. *n* = 3 for each cell type (biological replicates). *n* = 2-3 wells for each condition (technical replicates).

### 2.16 Statistical analyses

Statistical analyses and graphs were produced using GraphPad Prism (version 9.1). Normality tests were performed according to Shapiro-Wilk tests (*α* = .05). All datasets passed normality tests and were analysed using unpaired two-tailed t-test (significance set to *p* < .05). Graphs were plotted as mean (*µ*) ± standard deviation (SD).

## 3 Results

### 3.1 CD146 is a marker of interfascicular cell populations

**FIGURE 1:**
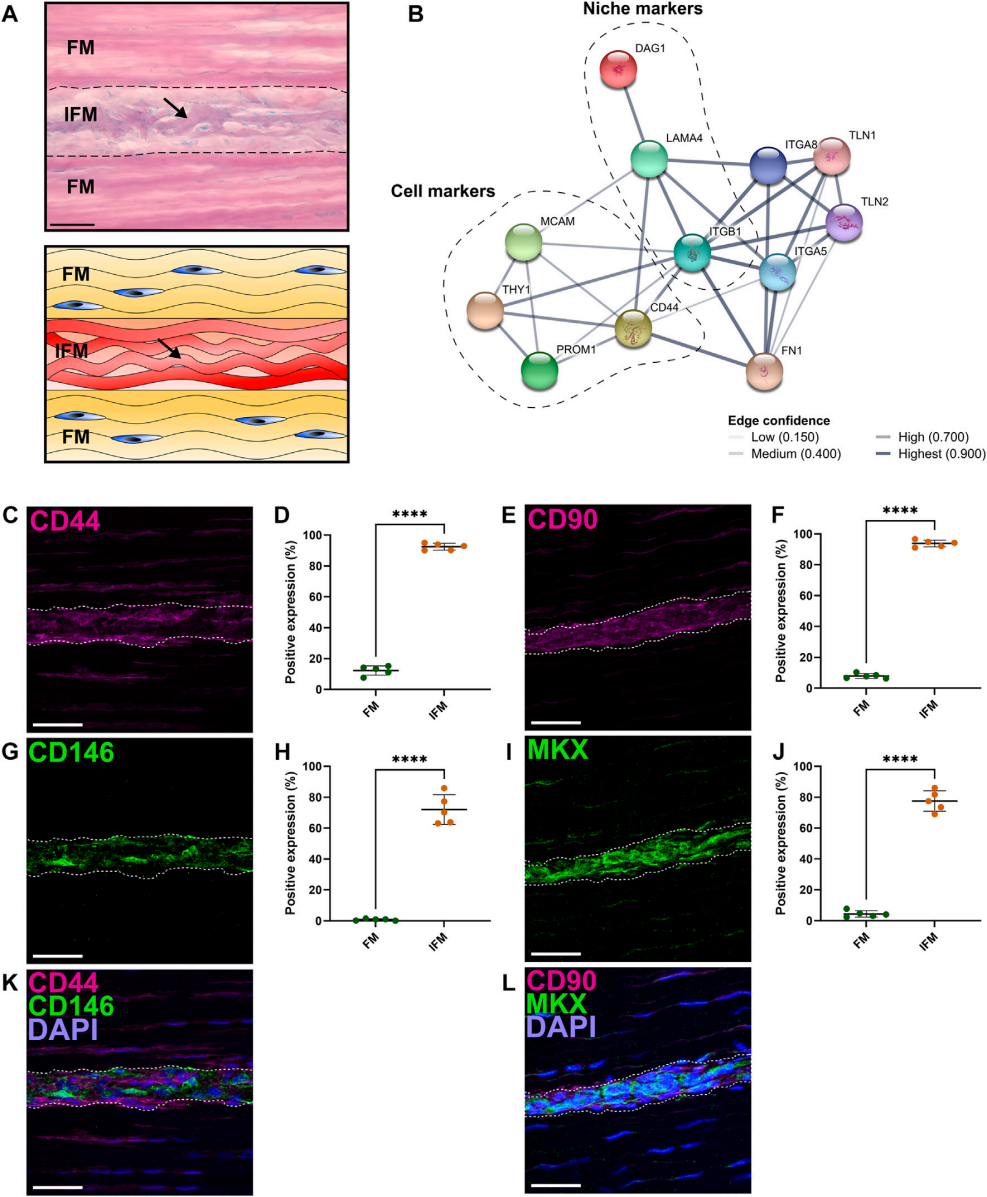
Analyses of regional differences in tendon cell marker expression demonstrated that CD146 is exclusively expressed by interfascicular cells within an interfascicular niche. **(A)** PAS-staining and schematic of SDFT sections highlighted mucin-rich basement membrane (arrow; purple, schematic; red) within the interfascicular matrix (IFM). Nuclei = blue. Scale bar = 50 µm. **(B)** STRING-predicted protein-protein interactions revealed potential targets for novel tendon cell populations using validated interfascicular niche markers CD146 and LAMA4. Interactions based on CD146 (MCAM) and LAMA4 demonstrated a protein neighborhood consisting of cell markers CD44, CD90 (THY1), CD133 (PROM1), as well as cell niche components such as ITGB1, DAG1 and FN1. **(C–J)** Image analyses comparing the positive labelling (area fraction; %) of longitudinal SDFT sections immunolabelled with CD44 **(C,D)**, CD90 **(E,F)**, CD146 **(G,H)** and MKX **(I,J)** overlayed with DAPI [blue = nuclei; **(K,L)**] in both fascicular matrix (FM) and IFM regions. The IFM is outlined by dotted lines. Scale bar = 50 µm. Biological replicates (*n*) = 5 per tendon region. Technical replicates = 3-4 per individual sample. Graphs were plotted as mean (µ) ± SD. Statistical significance: **** (*p* ≤ .0001).

**FIGURE 2:**
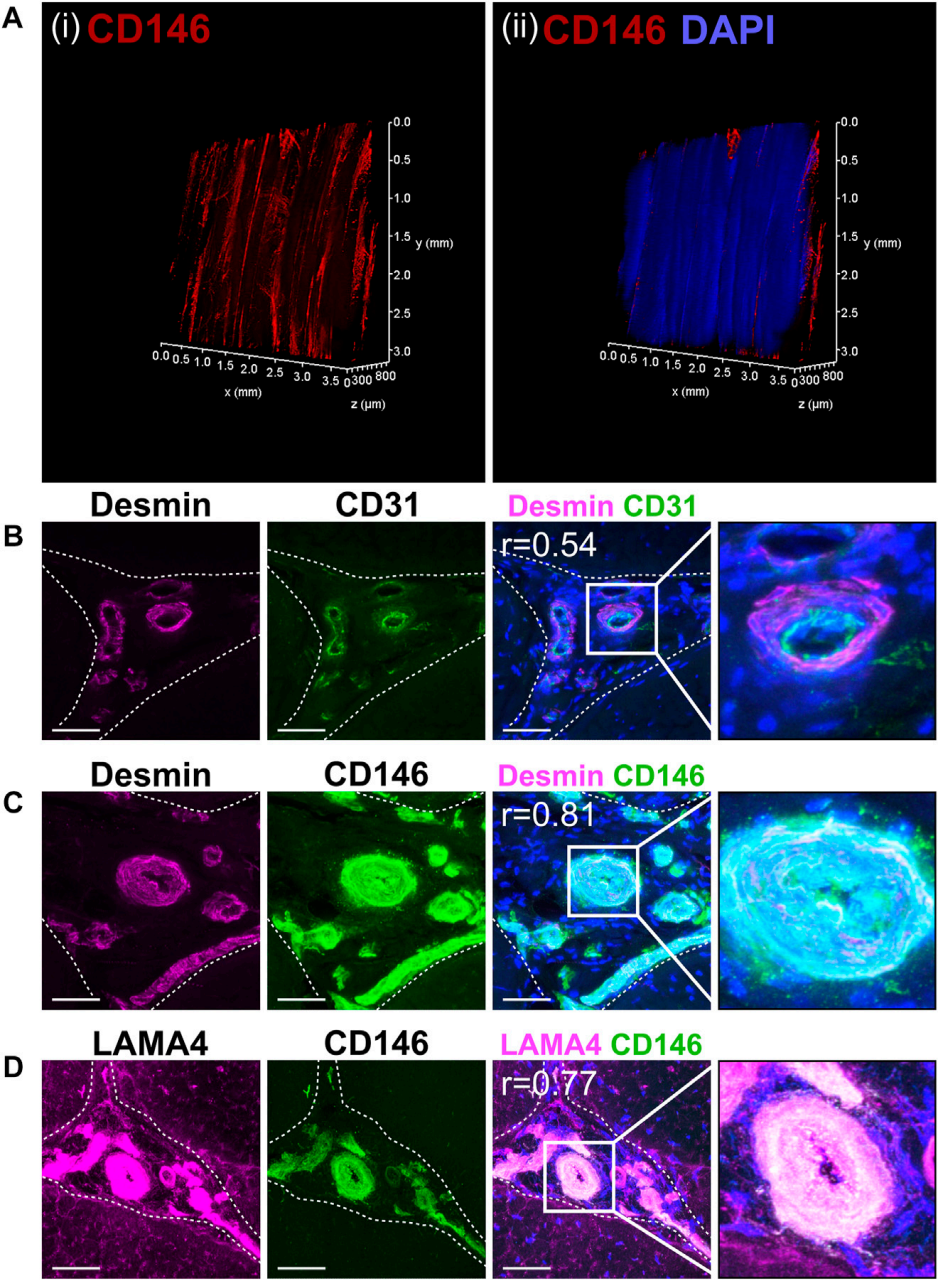
CD146 cell populations demarcated a vascular network indicative of a vascular cell niche within the interfascicular matrix. **(A)** 3D imaging of (i) CD146 and (ii) CD146 and nuclei (DAPI, blue) confirmed that the IFM was enriched with CD146 cell populations as part of an interconnected vessel network. Images of labelled transverse SDFT sections demonstrating colocalisation of Desmin with CD31 and CD146 **(B,C)** and CD146 with LAMA4 **(D)** indicating that CD146 represents a marker of interfascicular vascular cell populations resident within laminin-rich vessels. Nuclei = DAPI (blue). IFM is demarcated by dashed lines in transverse images. Scale bar = 50 µm. Images represent maximum projection of 30 µm section.

PAS staining demonstrated that the IFM contains mucin-rich basement membrane (Figure 1A). Using both CD146 and the IFM basement membrane marker LAMA4 in STRING predictions identified several potential interfascicular cell surface markers including CD44, CD90 (THY1) and CD133 (PROM1), as well as a broader network of interfascicular niche and basement membrane components, including dystroglycan 1 (DAG1), integrin subunit β1 (ITGB1) and fibronectin 1 (FN1) (Figure 1B). To validate these proposed interfascicular cell markers, fluorescent labelling of CD44, CD90, CD146 and MKX was quantified in both fascicular and interfascicular regions. All markers were enriched within IFM (72%–94% positive expression) and had significantly less expression within fascicles; fascicular CD146 expression was less than 1% whereas CD44, CD90 and MKX expression was between 4% and 15% (Figures 1C–J). Using 3D imaging of SDFT labelled with CD146, we identified an interfascicular network of vascular structures within which CD146 cells were localised (Figure 2A). The colocalisation of Desmin (mural cell marker) with CD31 (endothelial marker) and CD146 (pan-vascular marker), alongside LAMA4 (basement membrane; Figures 2B–D) observed in transverse sections confirmed that the structures were vascular, and often found in regions of IFM connecting 3 adjacent fascicles. Within IFM, we observed distinct CD31 endothelium surrounded by Desmin-rich layers, whilst CD146 and Desmin colocalised in smooth muscle and pericyte layers. Labelling of CD31, CD146 and Desmin was also confirmed in vascular layers of larger blood vessels within epitendinous regions (Supplementary Figure S5).

### 3.2 Tendon interfascicular matrix is enriched in an endothelial basement membrane

**FIGURE 3:**
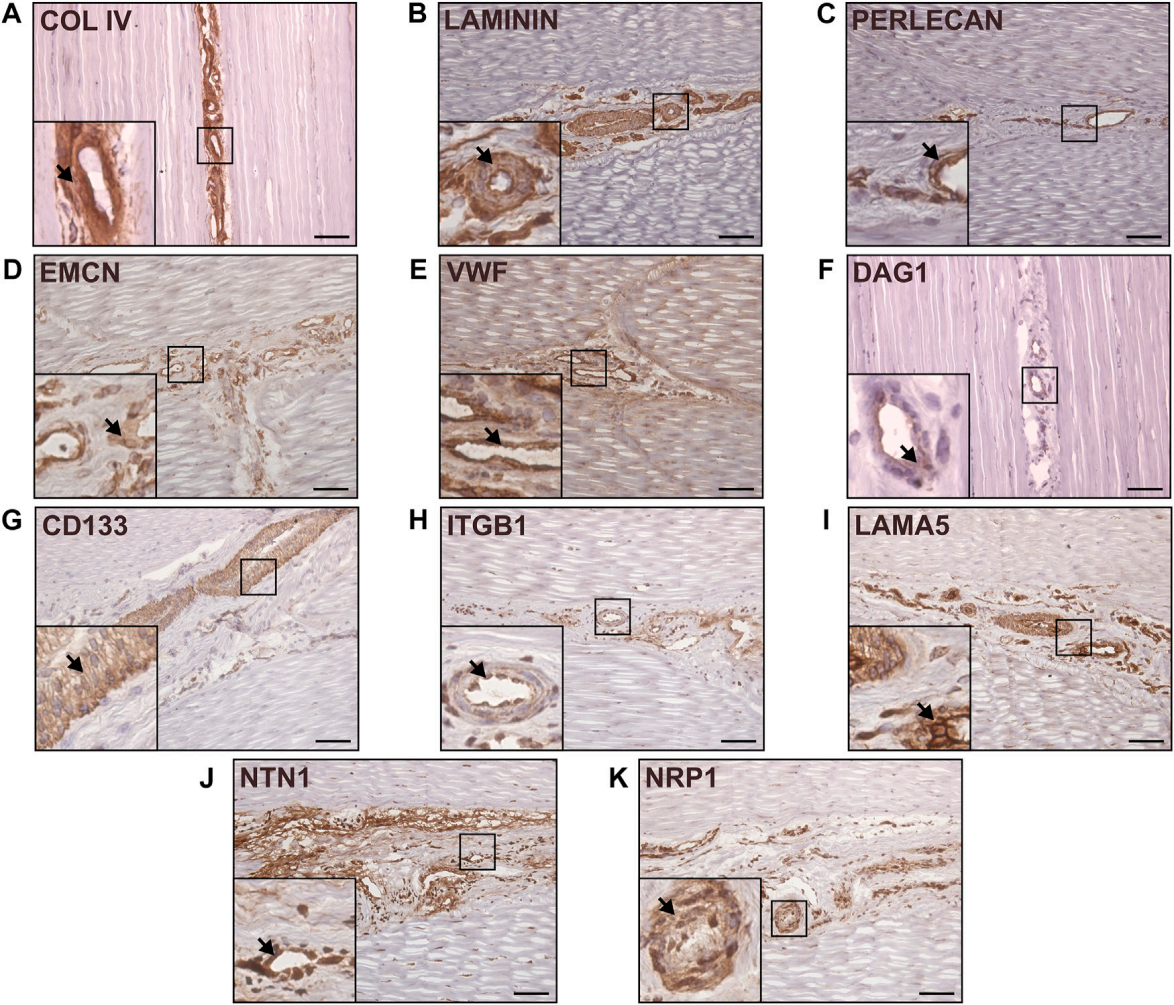
Canonical and network-predicted vascular and basement membrane components were enriched within the interfascicular matrix. Immunohistochemical labelling of longitudinal sections confirmed interfascicular expression of Type IV collagen **(A)**, full-length laminin **(B)**, Perlecan **(C)**, as well as vascular markers EMCN **(D)** and VWF **(E)**. Immunolabelling validation also confirmed enrichment within interfascicular vasculature with network-predicted markers DAG1 (IIH6; **(F)**, CD133 **(G)**, ITGB1 **(H)**, laminin isoform LAMA5 **(I)**, and angiogenic mediators NTN1 **(J)** and NRP1 **(K)**. Scale bar = 75 µm. Representative images shown.

To characterise the distribution of the major components of interfascicular basement membrane, we performed immunolabelling of basement membrane proteins in longitudinal tendon sections, including full-length laminin (pan-laminin), type IV collagen, and Perlecan (Figures 3A–C), all of which localised to the vasculature within IFM. Further labelling with endothelial markers endomucin (EMCN) and von Willebrand factor (VWF) demonstrated abundant expression within the IFM (Figures 3D, E). STRING predictions in *Equus caballus* identified canonical basement membrane components integrin β1 (ITGB1) and dystroglycan 1 (DAG1), as part of the CD146-LAMA4 interaction network. Hence, we performed labelling of *α*-dystroglycan (IIH6) and ITGB1 (Figures 3F, H), in addition to network-predicted cell surface marker CD133 (Figure 3G) with all three labelled abundantly within the IFM. As LAMA4/LAMA5 ratios are critical for basement membrane integrity (Galatenko et al., 2018), we also demonstrated labelling for laminin α5 (LAMA5) within the IFM (Figure 3I). We also examined other reported angiogenic mediators, Netrin-1 (NTN1); a reported ligand of CD146, and Neuropilin-1 (NRP1), both of which also localised to the IFM (Figures 3J, K). In addition to demonstrating the presence of these vascular and basement membrane markers in the IFM, our results highlight the variability in IFM vasculature and morphology in the mid-metarcarpal region of the SDFT, with a complex vascular network distinguishable in transverse and longitudinal sections, as well as 3D imaging (Figure 2).

### 3.3 Interfascicular CD146^+^ cells are a rare subpopulation requiring enrichment for *in vitro* isolation

**FIGURE 4:**
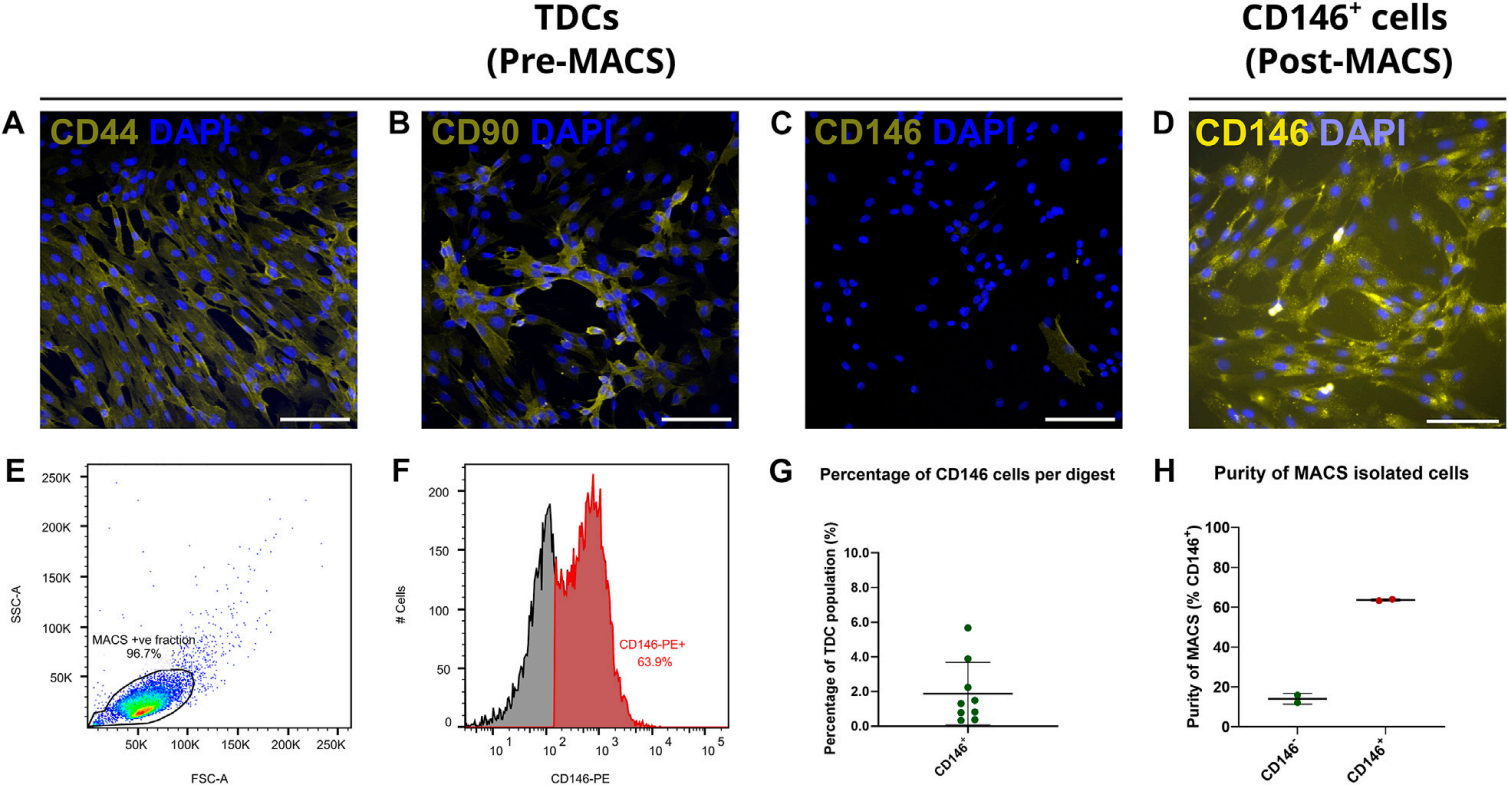
Immunocytochemical labelling confirms CD146^+^ cells are a rare subpopulation when expanded *in vitro,* and can be enriched *via* MACS. Tendon cell-surface markers CD44 **(A)**, CD90 **(B)** and CD146 **(C)** demonstrate that CD146 cells are a rare subpopulation amongst cultured TDCs (passage 1) when compared to cells expressing CD44 or CD90. *n* = 3 (biological replicates). Scale bar = 100 µm. Immunocytochemically labelled CD146 cells (D; passage 2) labelled with CD146 reaffirmed that expression persisted once expanded *in vitro*. *n* = 3 (biological replicates). Scale bar = 100 µm. Flow cytometry **(E,F)** confirmed CD146 expression in CD146^+^ cell populations. Total events = 10,000. **(G)** The percentage of CD146 cells (approx. 2%) yields by MACS from the original tendon-derived cell suspensions as determined by cell counting, reiterated the rarity of CD146 cells. *n* = 9 (biological replicates). Across MACS isolations **(H)**, flow cytometry confirms CD146 enrichment, albeit approximately 15% of cells in the CD146^−^ negative fraction were positive for CD146. *n* = 2 (biological replicates) per cell fraction. Graphs were plotted as mean (*µ*) ± SD.

Upon isolation from the SDFT, *in vitro* labelling of cell surface markers demonstrated that the majority of TDCs exhibited abundant CD44 and CD90 labelling and limited CD146 expression (Figures 4A–C). To study CD146 cells *in vitro*, we therefore developed a MACS procedure for the enrichment of CD146 cells. Immunocytochemistry of positively sorted CD146 cells confirmed enrichment for cells expressing CD146 (Figure 4D). A single application of MACS was able to yield CD146 cells with enrichment of approximately 64% as determined by flow cytometry (Figures 4E, F). Comparison of cell numbers pre and post MACS showed that approximately 2% of unsorted cells were CD146 positive (Figure 4G), providing further emphasis on the rarity of CD146 cell subpopulations and requirements for optimal enrichment procedures. However, some CD146 positive cells were detected in negative fractions (approx. 14%; Figure 4H).

### 3.4 Interfascicular CD146 cells have limited differentiation potential

**FIGURE 5:**
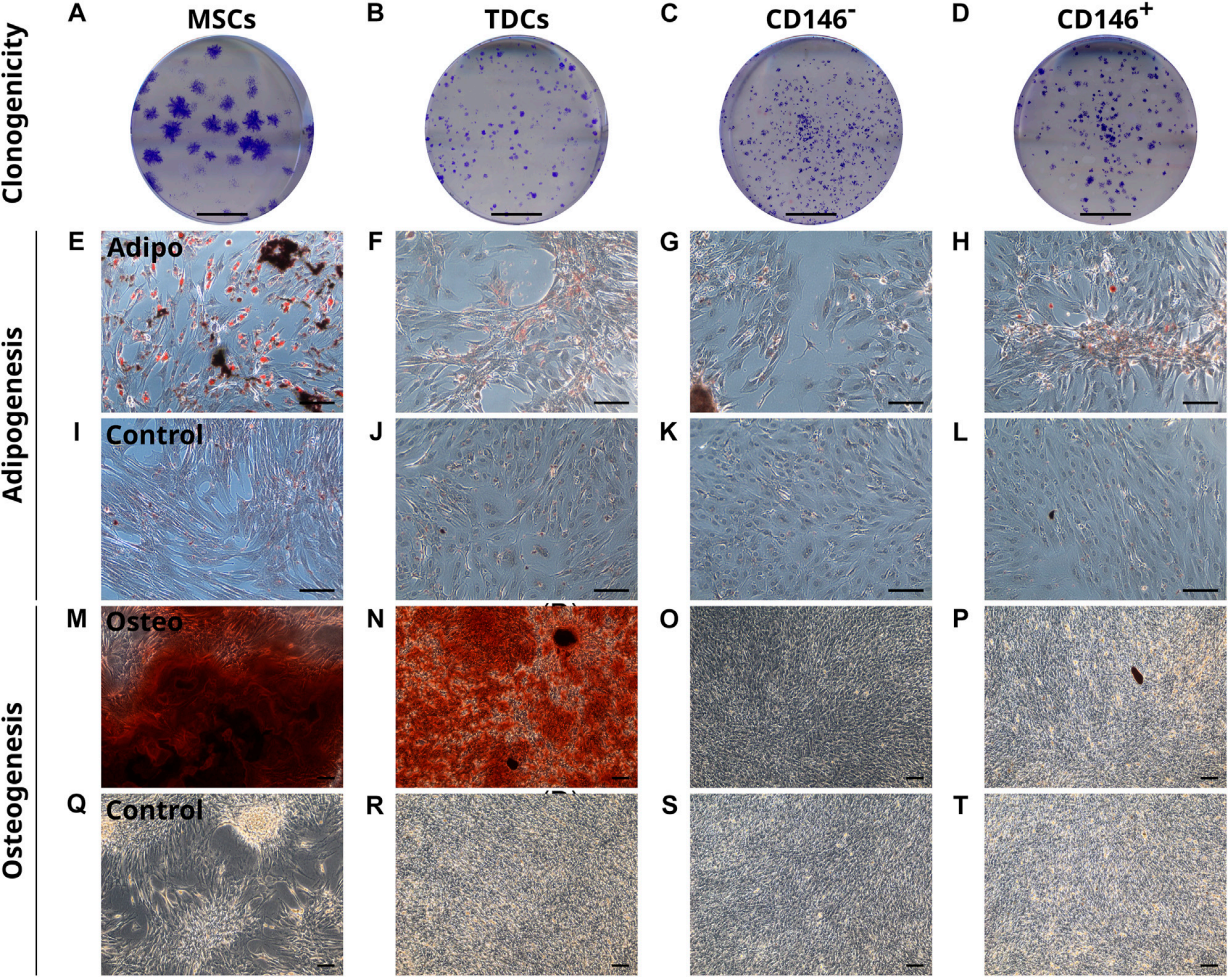
CD146^+^ cells exhibit limited clonogenicity and lineage potential. Representative images of colonies formed by MSCs, TDCs, CD146^-^ and CD146^+^ populations **(A–D)**. Scale bar = 1 cm. Oil Red O staining of MSCs, TDCs, CD146^-^ and CD146^+^ cells **(E–L)** under adipogenic conditions using StemPro® Adipogenesis differentiation media **(E–H)** and control conditions **(I–L)** demonstrate that TDCs, CD146^+^ and CD146^−^ cells produce a limited number of lipid vesicles. Lipid vesicles = red. *n* = 3 per cell type (biological replicates). *n* = 3 per condition (technical replicates). Scale bar = 100 µm. Alizarin Red S staining of MSCs, TDCs, CD146^-^ and CD146^+^ cells **(M–T)** under osteogenic conditions containing 2 mM DiP **(M–P)** and control conditions **(Q–T)** demonstrate that tendon cells exhibit limited mineralisation capacity when separated. Mineralised nodules = black. Calcium deposits = red. Unmineralised matrix = reflective/white. Scale bar = 100 µm. Images shown are representative of each condition.

To assess their clonogenicity and multi-lineage potential, unsorted TDCs, CD146^+^, CD146^−^ cells were subjected to clonogenic, osteogenic and adipogenic assays using MSCs as a positive control. CD146^+^ cells showed no enhanced clonogenicity compared to CD146-negative cells or heterogenous TDCs (Figures 5A–D). For adipogenesis, TDCs, CD146^+^ and CD146^−^ cells all showed limited adipogenic potential when stimulated (Figures 5E–H). Under osteogenic conditions, unsorted TDCs displayed extensive calcium deposition with some mineralised nodules present, however virtually no calcium deposition nor mineralisation was detected in either CD146^+^ and CD146^−^ sorted cell populations (Figures 5M–P).

## 4 Discussion

In this study, we have characterised CD146^+^ cell populations and their niche within the tendon IFM. We demonstrate that CD146^+^ cells exclusively localise to the IFM in healthy tendon, forming extensive interconnected 3D networks, comprising a niche containing vascular basement membrane and vascular-associated cells and proteins, several of which have been identified in the IFM for the first time. In contrast to our hypothesis that the IFM is a progenitor cell niche, CD146^+^ cells exhibited limited differentiation potential, indicating they are unlikely to be stem/progenitor cells, and are instead likely of vascular derivation.

The presence of CD146^+^ cells in tendon has been demonstrated previously; with immunolabelling of the human Achilles showing CD146 within the IFM (Yin et al., 2016). Our results support these findings. In addition, single-cell RNA sequencing of human tendon revealed three cell populations that express CD146; one of which was an endothelial population which co-expressed CD31 (Kendal et al., 2020). Furthermore, in single-cell analyses of mouse tendon, CD146^+^ tendon cells, identified as haematopoietic cells, represented around 9% of TDCs (De Micheli et al., 2020). In other tissues such as bone, CD31 and CD146 expression can be used to delineate endosteal and vascular populations which remodel the haematopoietic niche (Sacchetti et al., 2007; Tormin et al., 2011). Previous research from our group has highlighted CD31 as an IFM-localised vascular marker (Godinho et al., 2017) whilst other studies have reported Desmin as a pericyte and muscle cell marker (Chan-Ling et al., 2004; Piercy et al., 2007). The localisation of CD146 and CD31 with Desmin we report herein suggests that CD146^+^ delineates IFM endothelial and mural populations, whilst CD31 distinguishes endothelial cells from Desmin^+^ smooth muscle cells and pericytes. Indeed, the interconnected network of CD146 positivity detected demonstrates the presence of an interconnected IFM vascular network, which is likely continuous throughout the entire tendon. While studies have demonstrated vascularization of the IFM in both the SDFT and Achilles (Kraus-Hansen et al., 1992; Ahmed et al., 1998), the abundance and complexity of these vessels has not been appreciated previously. Further, epitendinous vessels (i.e., arteries and veins) are distinct when compared to interfascicular arterioles, capillaries or venules, given the observed differences in smooth muscle layers and whole vessel. Future studies are therefore required to improve classification of tendinous vasculature and the role of vasculature-associated cells in tendon homeostasis and repair.

In addition, our recent studies have established that CD146^+^ cells migrate to sites of injury in the rat Achilles tendon, which is accompanied by increased LAMA4 expression (Marr et al., 2021). In the current study, 3D imaging of the SDFT revealed an extensive interconnected network of CD146 labelling within the IFM; together these findings suggest that CD146^+^ cells are found both within, and separate from the vasculature. However it is yet to be established whether these are two distinct cell populations, or whether vascular-associated CD146 cells are able to migrate away from the vasculature. The interconnected network of CD146 forms structures which were similar those seen in 3D imaging of LAMA4 in SDFT (Marr et al., 2020). The colocalisation of CD146 and LAMA4 in the current study further reinforces the putative ligand-receptor interaction that CD146 and LAMA4 share, which has been demonstrated in previous studies (Ishikawa et al., 2014; Wragg et al., 2016). In chondrocytes, blocking of LAMA4 inhibited cluster formation, which is typical of pathological cartilage, and also resulted in downregulation of Claudin-1 (previously identified as a tendon IFM protein) and MMP3 (Fuerst et al., 2011; Moazedi-Fuerst et al., 2016). Recent studies have already established that loss of LAMA4 results in reduced CD146 cell expression and loss of basement membrane/niche maintenance in both mesenchymal and haematopoietic environments (Cai et al., 2022). Therefore, LAMA4 may act as a homing receptor for migrating interfascicular CD146^+^ tenocytes, however the chemokines that facilitate this are yet to be identified.

Here, we demonstrate that both LAMA4 and LAMA5 are abundant within the IFM niche, alongside other vascular components ITGB1, VWF, EMCN, NTN1 and NRP1. It has been shown previously that the early stage deletion of the laminin α4-chain is not functionally compensated for by other laminin chains leading to failed angiogenic development. Yet, in contrast, compensatory upregulation of LAMA5 as a result of LAMA4 loss results in a relatively milder vascular phenotype during postnatal maturation, which suggests that the balance between laminin subunits LAMA4/LAMA5 ratios is critical for maintaining a healthy vascular network and vascular niche (Thyboll et al., 2002). Given the abundant expression of both LAMA4 and LAMA5 found in our studies, it is likely that both chains and their full-length laminin isoforms 411 and 511 are essential to IFM endothelial basement membrane function, due to their previously reported role in shear-stress response and mechanotransduction (Di Russo et al., 2017; Beguin et al., 2020).

*In situ*, IFM cells were also positive for CD44 and CD90. Although their expression is likely acquired at later stages of differentiation and proliferation. These markers have been used to label putative stem/progenitor cell populations in tendon and other tissues (Leonardi et al., 2021). However, given that both markers were widely expressed throughout the IFM and fascicles, it is unlikely that they specifically label tendon stem cells in the equine SDFT and instead label several populations within tendon, including the tenocytes resident within fascicles. This assertion is supported by single-cell RNA sequencing data from the mouse Achilles tendon showing that both CD44 and CD90 are expressed by tenocytes and other tendon cell populations (De Micheli et al., 2020).

The identification of multiple vascular structures using markers of endothelial/vascular cell lineages demonstrates that the IFM houses a specialised vascular niche, rich in basement membrane proteins. This builds on our previous proteomics data showing enrichment of basement membrane proteins in the IFM, including perlecan, laminins and collagen type IV (Thorpe et al., 2016). The identification of perlecan-rich vascular networks in tendon IFM has major implications for the study of tendon. During development, perlecan is integral for tight packaging of interstitial tissues, which house vasculature, to ensure that maturation of endothelial tissues proceeds (Gustafsson et al., 2013). In addition, lymphangiogenesis within interstitial tissues is defined by the expression of perlecan and interstitial fluid flow (Rutkowski et al., 2006). In tendons, fascicular sliding may therefore be integral to IFM lymphatic and vascular remodelling. Moreover, VWF is likely to act as an endothelial cell ligand within the interfascicular basement membrane. The assembly of vascular basement membranes are regulated by β1-integrins and dystroglycans, and are typically formed of type IV collagens, proteoglycans such as perlecan, as well as vascular laminin isoforms comprised of LAMA4 and LAMA5 (Nikolova et al., 2006; Thomsen et al., 2017). Previous studies have reported vascular cell niches housing CD146-expressing stem/progenitor populations (Castrechini et al., 2010). Furthermore, the angiogenic capacity of CD146 is controlled by signalling molecules such as Netrin-1 and Neuropilin-1; both of which are critical for vascular cell patterning (Melani and Weinstein, 2010; Tu et al., 2015; Chen et al., 2018). It is notable that several of the above mentioned proteins, most of which are predicted to interact with CD146, localise to the IFM, indicating tethering of CD146 cells to a vascular basement membrane.

*In vitro*, tendon derived cells showed similar protein expression to that seen *in situ*, with abundant labelling of CD44 and CD90, and limited labelling for CD146 in only 2% of TDC. This is somewhat lower than the 9% of cells in the mouse Achilles that expressed CD146 as determined by single cell sequencing (De Micheli et al., 2020); this discrepancy may be explained by species-specific differences. The equine model is a highly relevant and well-accepted model for tendon research as the SDFT and human Achilles share similar function, structure and injury risk (Innes and Clegg, 2010; Patterson-Kane and Rich, 2014). Another explanation for this discrepancy in population proportions is the removal of the epitenon in the current study, which is known to house CD146^+^ cells (Marr et al., 2020). MACS was successfully employed to enrich CD146 populations, with approximately 65% of cells positive for CD146 post-sorting as determined by flow cytometry. This percentage is likely an under-representation, as CD146^+^ cells were detected using flow cytometry immediately post MACS-enrichment. This suggests that some CD146 antigens may still be bound to the magnetic label used during MACS, rendering them unavailable for binding to fluorescently tagged antibodies and hence non-detected by flow cytometry. Indeed, immunocytochemistry of CD146^+^ cells showed that virtually all cells labelled positively for CD146 post-MACS enrichment. However, a proportion of negatively selected cells expressed CD146 following cell sorting, likely due to a small number of CD146^+^ cells not binding to the column and therefore being eluted with the negative fraction. Enrichment could have been improved by additional rounds of sorting; however, this would have resulted in insufficient cell numbers for downstream experiments.

While previous studies have reported CD146 as a marker of mesenchymal stem cell lineages, tendon-derived CD146 populations exhibited similar clonogenicity to other TDCs, as well as limited differentiation potential. These findings agree with previous studies that demonstrated equine SDFT-derived TSPCs have limited clonogenicity and differentiation potential; this study utilised low density plating as opposed to cell sorting procedures to obtain TSPCs yet still failed to detect adipogenesis following stimulation (Williamson et al., 2015). In the current study, a limited number of lipid vesicles were however produced in adipogenic-induced TDCs, CD146^+^ and CD146^−^ cells. Unfortunately, we were unable to assess chondrogenesis after sorting due to the insufficient number of CD146^+^ cells for micromass survival during chondrogenic pellet induction. Together, these results indicate that CD146^+^ tendon-derived cells do not exhibit stem cell plasticity and instead CD146 is a pan-vascular marker in tendon, labelling both mural and endothelial cells. While the multipotency of pericytes has been demonstrated in a range of species (Esteves and Donadeu, 2018), other studies have shown that pericyte plasticity varies between tissue types, with some pericytes having limited differentiation potential (Herrmann et al., 2016). It is possible that, while tendon pericytes have a limited multipotency, they can differentiate down a tenogenic lineage, and indeed single cell sequencing data indicate that pericytes are a source of progenitor cells for adult tenocytes in murine tendon (De Micheli et al., 2020). However, as we did not assess tenogenesis in CD146 subpopulations, we are unable to confirm this and therefore future studies will need to fully characterize CD146 subpopulations. As tendon CD146^+^ populations have been shown to migrate to sites of injury, establishing further understanding of their local microenvironment, lineage origins, *in vitro* characteristics, and the effects of ageing will aid future research aimed at establishing if mobilising these populations can enhance intrinsic repair.

## 5 Conclusion

CD146 demarcates an IFM-specific cell population that reside in a niche rich in basement membrane and vascular proteins in tendons. Contrary to our hypothesis, CD146^+^ cells have limited clonogenicity and differentiation potential indicating they are unlikely to be stem/progenitor cells. Instead, co-localisation of Desmin with CD31 and CD146 indicates that CD146 is a pan-vascular marker within tendon. As previous studies have shown that CD146 cells migrate to sites of injury, establishing regenerative strategies that utilise endogenous tendon cell populations to promote intrinsic repair could act as a viable and effective method for improving healing responses and preventing tendon re-injury.

## Data Availability

The original contributions presented in the study are included in the article/Supplementary Material, further inquiries can be directed to the corresponding author.

## References

[B1] Ahmed I. M. Lagopoulos M. McConnell P. Soames R. W. Sefton G. K. (1998). Blood supply of the achilles tendon. J. Orthop. Res. 16 (5), 591–596. 10.1002/jor.1100160511 9820283

[B2] Alexander R. M. (1991). Energy-saving mechanisms in walking and running. J. Exp. Biol. 160 (1), 55–69. 10.1242/jeb.160.1.55 1960518

[B3] Alexander R. M. (2002). Tendon elasticity and muscle function. Comp. Biochem. Physiol. A Mol. Integr. Physiol. 133 (4), 1001–1011. 10.1016/s1095-6433(02)00143-5 12485689

[B4] Anderson D. M. Arredondo J. Hahn K. Valente G. Martin J. F. Wilson-Rawls J. (2006). Mohawk is a novel homeobox gene expressed in the developing mouse embryo. Dev. Dyn. 235 (3), 792–801. 10.1002/dvdy.20671 16408284

[B5] Autengruber A. Gereke M. Hansen G. Hennig C. Bruder D. (2012). Impact of enzymatic tissue disintegration on the level of surface molecule expression and immune cell function. Eur. J. Microbiol. Immunol. (Bp) 2 (2), 112–120. 10.1556/EuJMI.2.2012.2.3 24672679 PMC3956959

[B6] Barnes M. J. (1975). Function of ascorbic acid in collagen metabolism. Ann. N. Y. Acad. Sci. 258 (1), 264–277. 10.1111/j.1749-6632.1975.tb29287.x 173224

[B7] Beguin E. P. Janssen E. F. J. Hoogenboezem M. Meijer A. B. Hoogendijk A. J. van den Biggelaar M. (2020). Flow-induced reorganization of laminin-integrin networks within the endothelial basement membrane uncovered by proteomics. Mol. Cell. Proteomics 19 (7), 1179–1192. 10.1074/mcp.RA120.001964 32332107 PMC7338090

[B8] Benjamin M. Kaiser E. Milz S. (2008). Structure-function relationships in tendons: A review. J. Anat. 212 (3), 211–228. 10.1111/j.1469-7580.2008.00864.x 18304204 PMC2408985

[B9] Bi Y. Ehirchiou D. Kilts T. M. Inkson C. A. Embree M. C. Sonoyama W. (2007). Identification of tendon stem/progenitor cells and the role of the extracellular matrix in their niche. Nat. Med. 13 (10), 1219–1227. 10.1038/nm1630 17828274

[B10] Biewener A. A. (1998). Muscle-tendon stresses and elastic energy storage during locomotion in the horse. Comp. Biochem. Physiol. B Biochem. Mol. Biol. 120 (1), 73–87. 10.1016/s0305-0491(98)00024-8 9787779

[B11] CaiH.KondoM.SandhowL.XiaoP.JohanssonA. S.SasakiT. (2022). Critical role of Lama4 for hematopoiesis regeneration and acute myeloid leukemia progression. Blood 139 (20), 3040–3057. 10.1182/blood.2021011510 34958665

[B12] Castrechini N. M. Murthi P. Gude N. M. Erwich J. J. H. M. Gronthos S. Zannettino A. (2010). Mesenchymal stem cells in human placental chorionic villi reside in a vascular Niche. Placenta 31 (3), 203–212. 10.1016/j.placenta.2009.12.006 20060164

[B13] Chan-Ling T. Page M. P. Gardiner T. Baxter L. Rosinova E. Hughes S. (2004). Desmin ensheathment ratio as an indicator of vessel stability: Evidence in normal development and in retinopathy of prematurity. Am. J. Pathology 165 (4), 1301–1313. 10.1016/s0002-9440(10)63389-5 PMC1618638 15466395

[B14] Chen J. Luo Y. Huang H. Wu S. Feng J. Zhang J. (2018). CD146 is essential for PDGFRβ-induced pericyte recruitment. Protein & Cell. 9 (8), 743–747. 10.1007/s13238-017-0484-5 29039032 PMC6053352

[B15] Dakin S. G. Werling D. Hibbert A. Abayasekara D. R. Young N. J. Smith R. K. (2012). Macrophage sub-populations and the lipoxin A4 receptor implicate active inflammation during equine tendon repair. PLoS One 7 (2), e32333. 10.1371/journal.pone.0032333 22384219 PMC3284560

[B16] De Micheli A. J. Swanson J. B. Disser N. P. Martinez L. M. Walker N. R. Oliver D. J. (2020). Single-cell transcriptomic analysis identifies extensive heterogeneity in the cellular composition of mouse Achilles tendons. Am. J. Physiol. Cell. Physiol. 319 (5), C885–C894. 10.1152/ajpcell.00372.2020 32877217 PMC7701267

[B17] Di Russo J. Luik A. L. Yousif L. Budny S. Oberleithner H. Hofschroer V. (2017). Endothelial basement membrane laminin 511 is essential for shear stress response. EMBO J. 36 (2), 183–201. 10.15252/embj.201694756 27940654 PMC5239996

[B18] Esteves C. L. Donadeu F. X. (2018). Pericytes and their potential in regenerative medicine across species. Cytom. Part A 93 (1), 50–59. 10.1002/cyto.a.23243 28941046

[B19] Evans N. D. Oreffo R. O. C. Healy E. Thurner P. J. Man Y. H. (2013). Epithelial mechanobiology, skin wound healing, and the stem cell niche. J. Mech. Behav. Biomed. Mater. 28, 397–409. 10.1016/j.jmbbm.2013.04.023 23746929

[B20] Fuerst F. C. Gruber G. Stradner M. H. Jones J. C. Kremser M. L. Angerer H. (2011). Regulation of MMP3 by laminin alpha 4 in human osteoarthritic cartilage. Scand. J. Rheumatol. 40 (6), 494–496. 10.3109/03009742.2011.605392 22150225 PMC4667954

[B21] Galatenko V. V. Maltseva D. V. Galatenko A. V. Rodin S. Tonevitsky A. G. (2018). Cumulative prognostic power of laminin genes in colorectal cancer. BMC Med. Genomics 11 (1), 9. 10.1186/s12920-018-0332-3 29504916 PMC5836818

[B22] Garvican E. R. Salavati M. Smith R. K. W. Dudhia J. (2017). Exposure of a tendon extracellular matrix to synovial fluid triggers endogenous and engrafted cell death: A mechanism for failed healing of intrathecal tendon injuries. Connect. Tissue Res. 58 (5), 438–446. 10.1080/03008207.2016.1245726 27726447

[B23] Godinho M. S. C. Thorpe C. T. Greenwald S. E. Screen H. R. C. (2017). Elastin is localised to the interfascicular matrix of energy storing tendons and becomes increasingly disorganised with ageing. Sci. Rep. 7 (1), 9713–9811. 10.1038/s41598-017-09995-4 28855560 PMC5577209

[B24] Godwin E. E. Young N. J. Dudhia J. Beamish I. C. Smith R. K. (2012). Implantation of bone marrow-derived mesenchymal stem cells demonstrates improved outcome in horses with overstrain injury of the superficial digital flexor tendon. Equine Vet. J. 44 (1), 25–32. 10.1111/j.2042-3306.2011.00363.x 21615465

[B25] Gumucio J. P. Schonk M. M. Kharaz Y. A. Comerford E. Mendias C. L. (2020). Scleraxis is required for the growth of adult tendons in response to mechanical loading. JCI Insight 5 (13), e138295. 10.1172/jci.insight.138295 32463804 PMC7406294

[B26] Gustafsson E. Almonte-Becerril M. Bloch W. Costell M. (2013). Perlecan maintains microvessel integrity *in vivo* and modulates their formation *in vitro* . PLoS One 8 (1), e53715. 10.1371/journal.pone.0053715 23320101 PMC3540034

[B27] Handsfield G. G. Slane L. C. Screen H. R. C. (2016). Nomenclature of the tendon hierarchy: An overview of inconsistent terminology and a proposed size-based naming scheme with terminology for multi-muscle tendons. J. Biomech. 49 (13), 3122–3124. 10.1016/j.jbiomech.2016.06.028 27421207

[B28] Herrmann M. Bara J. J. Sprecher C. M. Menzel U. Jalowiec J. M. Osinga R. (2016). Pericyte plasticity - comparative investigation of the angiogenic and multilineage potential of pericytes from different human tissues. Eur. Cell. Mater 31, 236–249. 10.22203/ecm.v031a16 27062725

[B29] Horwitz E. M. Le Blanc K. Dominici M. Mueller I. Slaper-Cortenbach I. Marini F. C. (2005). Clarification of the nomenclature for MSC: The international society for cellular therapy position statement. Cytotherapy 7 (5), 393–395. 10.1080/14653240500319234 16236628

[B30] Innes J. F. Clegg P. (2010). Comparative rheumatology: What can be learnt from naturally occurring musculoskeletal disorders in domestic animals? Rheumatol. Oxf. 49 (6), 1030–1039. 10.1093/rheumatology/kep465 20176567

[B31] Ishikawa T. Wondimu Z. Oikawa Y. Gentilcore G. Kiessling R. Egyhazi Brage S. (2014). Laminins 411 and 421 differentially promote tumor cell migration via α6β1 integrin and MCAM (CD146). Matrix Biol. 38, 69–83. 10.1016/j.matbio.2014.06.002 24951930

[B32] Ivanovska I. L. Shin J. W. Swift J. Discher D. E. (2015). Stem cell mechanobiology: Diverse lessons from bone marrow. Trends Cell. Biol. 25 (9), 523–532. 10.1016/j.tcb.2015.04.003 26045259 PMC4555184

[B33] Johnson J. P. Rothbacher U. Sers C. (1993). The progression associated antigen MUC18: A unique member of the immunoglobulin supergene family. Melanoma Res. 3 (5), 337–340. 10.1097/00008390-199310000-00006 8292890

[B34] Kaltz N. Ringe J. Holzwarth C. Charbord P. Niemeyer M. Jacobs V. R. (2010). Novel markers of mesenchymal stem cells defined by genome-wide gene expression analysis of stromal cells from different sources. Exp. Cell. Res. 316 (16), 2609–2617. 10.1016/j.yexcr.2010.06.002 20599957

[B35] Kannus P. (2000). Structure of the tendon connective tissue. Scand. J. Med. Sci. Sports 10 (6), 312–320. 10.1034/j.1600-0838.2000.010006312.x 11085557

[B36] Kendal A. R. Layton T. Al-Mossawi H. Appleton L. Dakin S. Brown R. (2020). Multi-omic single cell analysis resolves novel stromal cell populations in healthy and diseased human tendon. Sci. Rep. 10 (1), 13939. 10.1038/s41598-020-70786-5 32883960 PMC7471282

[B37] Kimura W. Machii M. Xue X. Sultana N. Hikosaka K. Sharkar M. T. (2011). Irxl1 mutant mice show reduced tendon differentiation and no patterning defects in musculoskeletal system development. Genesis 49 (1), 2–9. 10.1002/dvg.20688 21254332

[B38] Kraus-Hansen A. E. Fackelman G. E. Becker C. Williams R. M. Pipers F. S. (1992). Preliminary studies on the vascular anatomy of the equine superficial digital flexor tendon. Equine Veterinary J. 24 (1), 46–51. 10.1111/j.2042-3306.1992.tb02778.x 1555540

[B39] Leonardi E. A. Xiao M. Murray I. R. Robinson W. H. Abrams G. D. (2021). Tendon-derived progenitor cells with multilineage potential are present within human patellar tendon. Orthop. J. Sports Med. 9 (8), 23259671211023452. 10.1177/23259671211023452 34435068 PMC8381435

[B40] Marr N. Hopkinson M. Hibbert A. P. Pitsillides A. A. Thorpe C. T. (2020). Bimodal whole-mount imaging of tendon using confocal microscopy and X-ray micro-computed tomography. Biol. Proced. Online 22 (1), 13. 10.1186/s12575-020-00126-4 32624710 PMC7329428

[B41] Marr N. Meeson R. Kelly E. F. Fang Y. Peffers M. J. Pitsillides A. A. (2021). CD146 delineates an interfascicular cell sub-population in tendon that is recruited during injury through its ligand laminin-α4. Int. J. Mol. Sci. 22 (18), 9729. 10.3390/ijms22189729 34575887 PMC8472220

[B42] Melani M. Weinstein B. M. (2010). Common factors regulating patterning of the nervous and vascular systems. Annu. Rev. Cell. Dev. Biol. 26 (1), 639–665. 10.1146/annurev.cellbio.093008.093324 19575651

[B43] Moazedi-Fuerst F. C. Gruber G. Stradner M. H. Guidolin D. Jones J. C. Bodo K. (2016). Effect of Laminin-A4 inhibition on cluster formation of human osteoarthritic chondrocytes. J. Orthop. Res. 34 (3), 419–426. 10.1002/jor.23036 26295200 PMC5727909

[B44] Morath I. Hartmann T. N. Orian-Rousseau V. (2016). CD44: More than a mere stem cell marker. Int. J. Biochem. Cell. Biol. 81, 166–173. 10.1016/j.biocel.2016.09.009 27640754

[B45] Nikolova G. Jabs N. Konstantinova I. Domogatskaya A. Tryggvason K. Sorokin L. (2006). The vascular basement membrane: A niche for insulin gene expression and β cell proliferation. Dev. Cell. 10 (3), 397–405. 10.1016/j.devcel.2006.01.015 16516842

[B46] Patel J. J. Bourne L. E. Davies B. K. Arnett T. R. MacRae V. E. Wheeler-Jones C. P. D. (2019). Differing calcification processes in cultured vascular smooth muscle cells and osteoblasts. Exp. Cell. Res. 380 (1), 100–113. 10.1016/j.yexcr.2019.04.020 31004580 PMC6520648

[B47] Patterson-Kane J. C. Rich T. (2014). Achilles tendon injuries in elite athletes: Lessons in pathophysiology from their equine counterparts. ILAR J. 55 (1), 86–99. 10.1093/ilar/ilu004 24936032

[B48] Piercy R. J. Zhou H. Feng L. Pombo A. Muntoni F. Brown S. C. (2007). Desmin immunolocalisation in autosomal dominant Emery-Dreifuss muscular dystrophy. Neuromuscul. Disord. 17 (4), 297–305. 10.1016/j.nmd.2007.01.003 17329105

[B49] Richardson L. E. Dudhia J. Clegg P. D. Smith R. (2007). Stem cells in veterinary medicine--attempts at regenerating equine tendon after injury. Trends Biotechnol. 25 (9), 409–416. 10.1016/j.tibtech.2007.07.009 17692415

[B50] Robey P. G. Termine J. D. (1985). Human bone cells*in vitro* . Calcif. Tissue Int. 37 (5), 453–460. 10.1007/BF02557826 2998572

[B51] Russell K. C. Phinney D. G. Lacey M. R. Barrilleaux B. L. Meyertholen K. E. O'Connor K. C. (2010). *In vitro* high-capacity assay to quantify the clonal heterogeneity in trilineage potential of mesenchymal stem cells reveals a complex hierarchy of lineage commitment. Stem Cells 28 (4), 788–798. 10.1002/stem.312 20127798

[B52] Rutkowski J. M. Boardman K. C. Swartz M. A. (2006). Characterization of lymphangiogenesis in a model of adult skin regeneration. Am. J. Physiol. Heart Circ. Physiol. 291 (3), H1402–H1410. 10.1152/ajpheart.00038.2006 16648194 PMC2751590

[B53] Sacchetti B. Funari A. Michienzi S. Di Cesare S. Piersanti S. Saggio I. (2007). Self-renewing osteoprogenitors in bone marrow sinusoids can organize a hematopoietic microenvironment. Cell. 131 (2), 324–336. 10.1016/j.cell.2007.08.025 17956733

[B54] Schindelin J. Arganda-Carreras I. Frise E. Kaynig V. Longair M. Pietzsch T. (2012). Fiji: An open-source platform for biological-image analysis. Nat. Methods 9 (7), 676–682. 10.1038/nmeth.2019 22743772 PMC3855844

[B55] Schlagbauer-Wadl H. Jansen B. Muller M. Polterauer P. Wolff K. Eichler H. G. (1999). Influence of MUC18/MCAM/CD146 expression on human melanoma growth and metastasis in SCID mice. Int. J. Cancer 81 (6), 951–955. 10.1002/(sici)1097-0215(19990611)81:6<951::aid-ijc18>3.0.co;2-v 10362144

[B56] Schrage A. Loddenkemper C. Erben U. Lauer U. Hausdorf G. Jungblut P. R. (2008). Murine CD146 is widely expressed on endothelial cells and is recognized by the monoclonal antibody ME-9F1. Histochem Cell. Biol. 129 (4), 441–451. 10.1007/s00418-008-0379-x 18214516 PMC2756363

[B57] Schweitzer R. Chyung J. H. Murtaugh L. C. Brent A. E. Rosen V. Olson E. N. (2001). Analysis of the tendon cell fate using Scleraxis, a specific marker for tendons and ligaments. Development 128 (19), 3855–3866. 10.1242/dev.128.19.3855 11585810

[B58] Sers C. Kirsch K. Rothbacher U. Riethmuller G. Johnson J. P. (1993). Genomic organization of the melanoma-associated glycoprotein MUC18: Implications for the evolution of the immunoglobulin domains. Proc. Natl. Acad. Sci. U. S. A. 90 (18), 8514–8518. 10.1073/pnas.90.18.8514 8378324 PMC47387

[B59] Shih I. M. (1999). The role of CD146 (Mel-CAM) in biology and pathology. J. Pathol. 189 (1), 4–11. 10.1002/(SICI)1096-9896(199909)189:1<4::AID-PATH332>3.0.CO;2-P 10451481

[B60] Smith L. R. Cho S. Discher D. E. (2018). Stem cell differentiation is regulated by extracellular matrix mechanics. Physiology 33 (1), 16–25. 10.1152/physiol.00026.2017 29212889 PMC5866410

[B61] Szklarczyk D. Morris J. H. Cook H. Kuhn M. Wyder S. Simonovic M. (2017). The STRING database in 2017: Quality-controlled protein-protein association networks, made broadly accessible. Nucleic Acids Res. 45 (D1), D362–D368. 10.1093/nar/gkw937 27924014 PMC5210637

[B62] Taylor S. E. Shah M. Orriss I. R. (2014). Generation of rodent and human osteoblasts. Bonekey Rep. 3, 585. 10.1038/bonekey.2014.80 25396049 PMC4230189

[B63] Thomsen M. S. Routhe L. J. Moos T. (2017). The vascular basement membrane in the healthy and pathological brain. J. Cereb. Blood Flow Metabolism 37 (10), 3300–3317. 10.1177/0271678x17722436 PMC5624399 28753105

[B64] Thorpe C. T. Godinho M. S. C. Riley G. P. Birch H. L. Clegg P. D. Screen H. R. C. (2015). The interfascicular matrix enables fascicle sliding and recovery in tendon, and behaves more elastically in energy storing tendons. J. Mech. Behav. Biomed. Mater 52, 85–94. 10.1016/j.jmbbm.2015.04.009 25958330 PMC4655227

[B65] Thorpe C. T. Peffers M. J. Simpson D. Halliwell E. Screen H. R. Clegg P. D. (2016). Anatomical heterogeneity of tendon: Fascicular and interfascicular tendon compartments have distinct proteomic composition. Sci. Rep. 6 (1), 20455. 10.1038/srep20455 26842662 PMC4740843

[B66] Thorpe C. T. Screen H. R. C. (2016b). “Tendon structure and composition,” in Metabolic influences on risk for tendon disorders. Editors Ackermann P. W. Hart D. A. (Cham: Springer International Publishing), 3–10.

[B67] Thorpe C. T. Screen H. R. (2016a). Tendon structure and composition. Adv. Exp. Med. Biol. 920, 3–10. 10.1007/978-3-319-33943-6_1 27535244

[B68] Thyboll J. Kortesmaa J. Cao R. Soininen R. Wang L. Iivanainen A. (2002). Deletion of the laminin alpha4 chain leads to impaired microvessel maturation. Mol. Cell. Biol. 22 (4), 1194–1202. 10.1128/MCB.22.4.1194-1202.2002 11809810 PMC134646

[B69] Tormin A. Li O. Brune J. C. Walsh S. Schutz B. Ehinger M. (2011). CD146 expression on primary nonhematopoietic bone marrow stem cells is correlated with *in situ* localization. Blood 117 (19), 5067–5077. 10.1182/blood-2010-08-304287 21415267 PMC3109533

[B70] Tu T. Zhang C. Yan H. Luo Y. Kong R. Wen P. (2015). CD146 acts as a novel receptor for netrin-1 in promoting angiogenesis and vascular development. Cell. Res. 25 (3), 275–287. 10.1038/cr.2015.15 25656845 PMC4349246

[B71] Webbon P. M. (1977). A post mortem study of equine digital flexor tendons. Equine Vet. J. 9 (2), 61–67. 10.1111/j.2042-3306.1977.tb03981.x 862604

[B72] Williamson K. A. Lee K. J. Humphreys W. J. Comerford E. J. Clegg P. D. Canty-Laird E. G. (2015). Restricted differentiation potential of progenitor cell populations obtained from the equine superficial digital flexor tendon (SDFT). J. Orthop. Res. 33 (6), 849–858. 10.1002/jor.22891 25877997 PMC4657492

[B73] Wragg J. W. Finnity J. P. Anderson J. A. Ferguson H. J. Porfiri E. Bhatt R. I. (2016). MCAM and LAMA4 are highly enriched in tumor blood vessels of renal cell carcinoma and predict patient outcome. Cancer Res. 76 (8), 2314–2326. 10.1158/0008-5472.CAN-15-1364 26921326 PMC4875769

[B74] Yin Z. Hu J. J. Yang L. Zheng Z. F. An C. R. Wu B. B. (2016). Single-cell analysis reveals a nestin(+) tendon stem/progenitor cell population with strong tenogenic potentiality. Sci. Adv. 2 (11), e1600874. 10.1126/sciadv.1600874 28138519 PMC5262457

