# Supplementary material for: The tendon interfascicular basement membrane provides a vascular niche for CD146+ cell subpopulations
Source: Front Cell Dev Biol. 2023 Jan 9;10:1094124. doi: 10.3389/fcell.2022.1094124 (PMC9869387; doi:10.3389/fcell.2022.1094124)
Supplement: Supplementary file 1 [file DataSheet1.docx]

# Supplementary Material

## Supplementary Figures


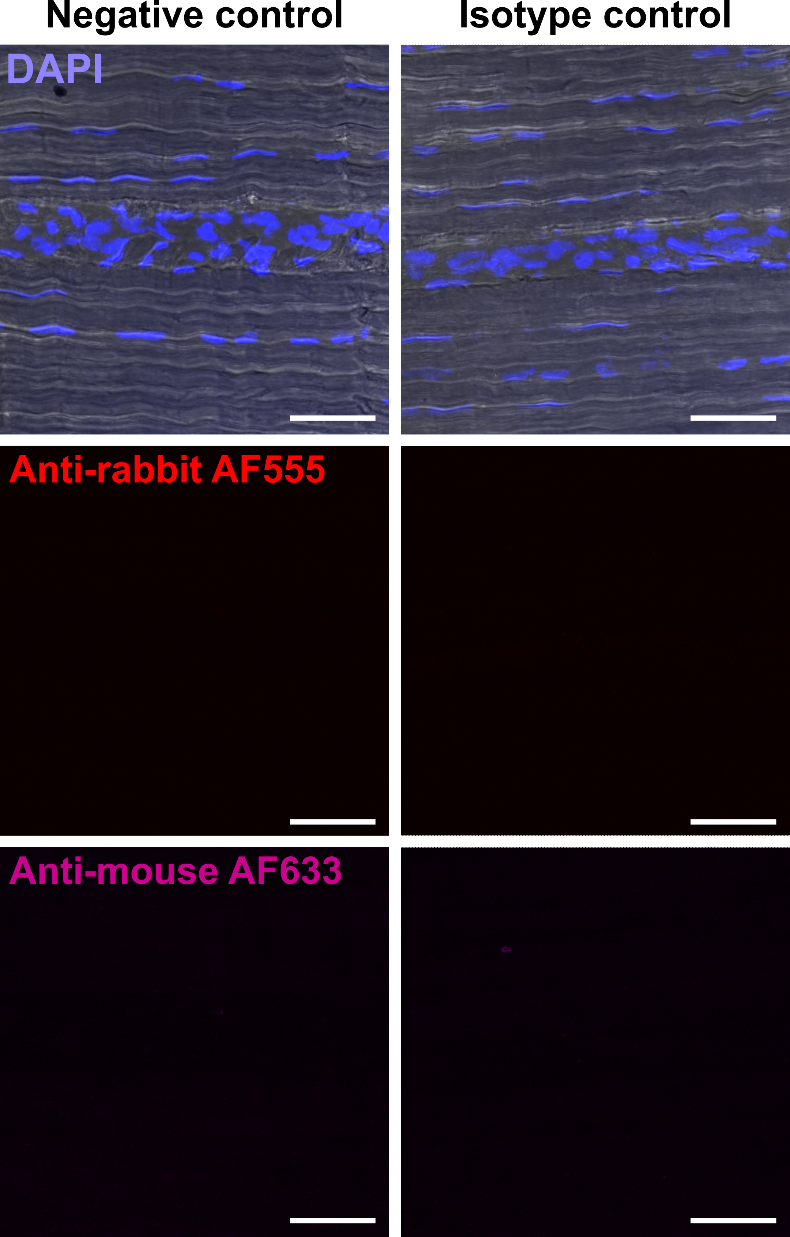


**Supplementary Figure S1.** Negative and isotype control labelling of SDFT tissues. Representative images of negative (left panel) control staining of SDFT tissue with goat anti-mouse Alexa Fluor® 633 and goat anti-rabbit IgG Alexa Fluor® 555 secondary antibodies applied only (1:100 for both). Isotype (right panel) matched control labelling was performed with rabbit IgG and mouse IgG isotype primary antibodies (1:100 for both) followed by incubation with secondary antibodies. Scale bar = 50 µm.


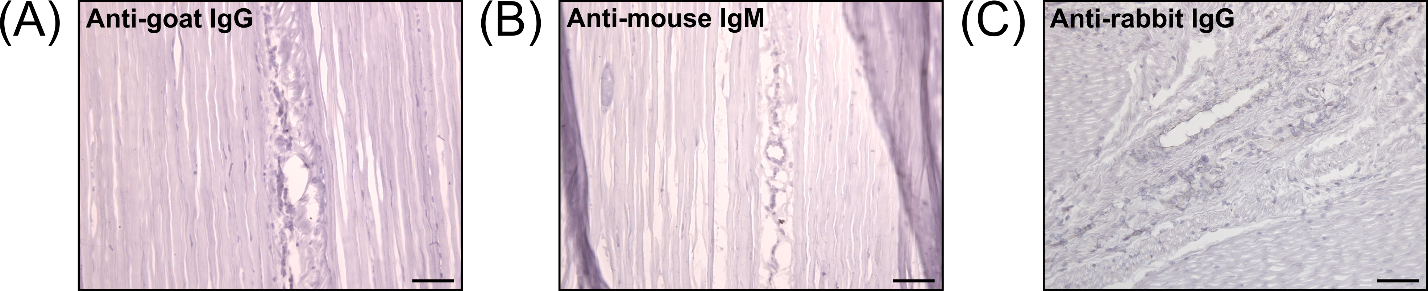


**Supplementary Figure S2.** Negative (secondary antibody) control staining for immunohistochemical labelling of SDFT. Longitudinal SDFT sections without primary antibodies labelled with (A) Dako rabbit anti-goat IgG secondary, and (B,C) EnVision peroxidase labelled polymer (conjugated to goat anti-mouse and goat anti-rabbit immunoglobulins). Scale bar = 75 µm.


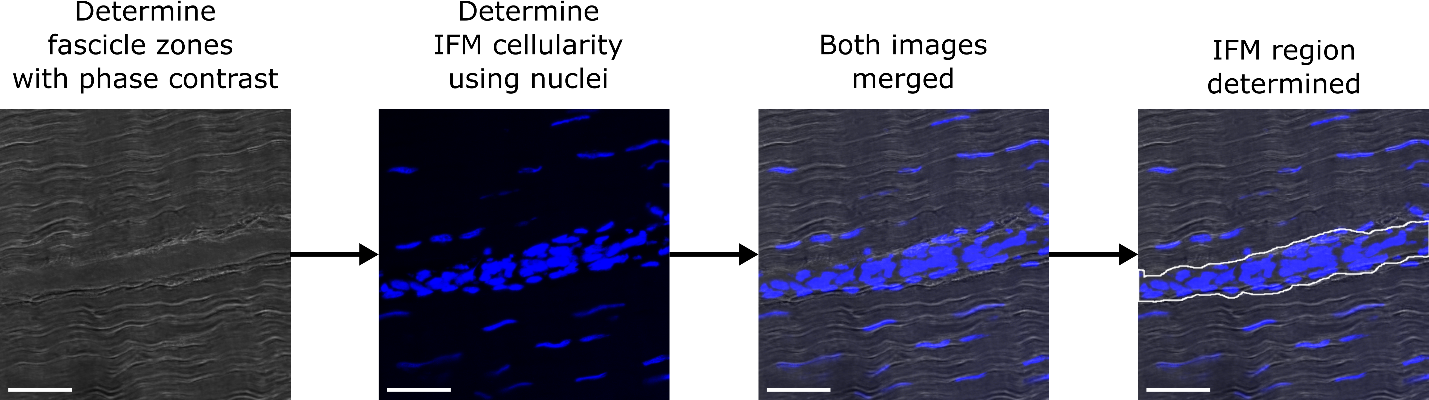


**Supplementary Figure S3.** Workflow for the determination of IFM and fascicular boundaries in longitudinal SDFT sections. Phase contrast imaging (grey) and nuclei (DAPI = blue) aid in identifying IFM and fascicle boundaries (dashed line). Scale bar = 50 µm.


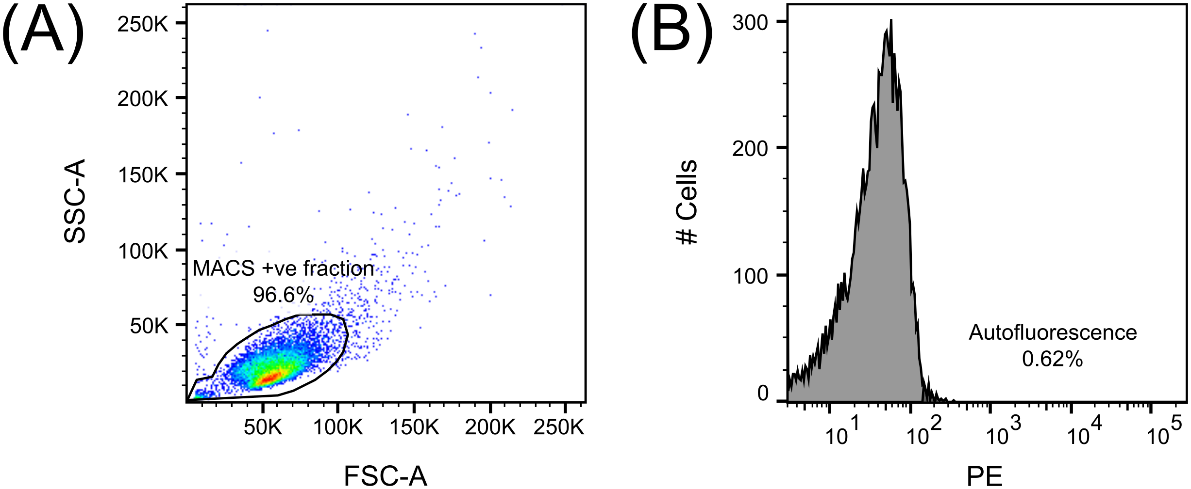


**Supplementary Figure S4.** Fluorescence minus one control (unstained) gating strategy for detection of CD146-PE labelling in MACS positive selection populations. Unstained MACS fractions were gated by size using SSC-A and FSC-A (A), and gated for PE fluorescence to discriminate positively and negatively labelled populations (B). The percentage of positive cells gated in unstained samples (i.e. autofluorescent cells) was subtracted from stained samples (i.e. experimental cells) to give an overall percentage of immunoreactivity.


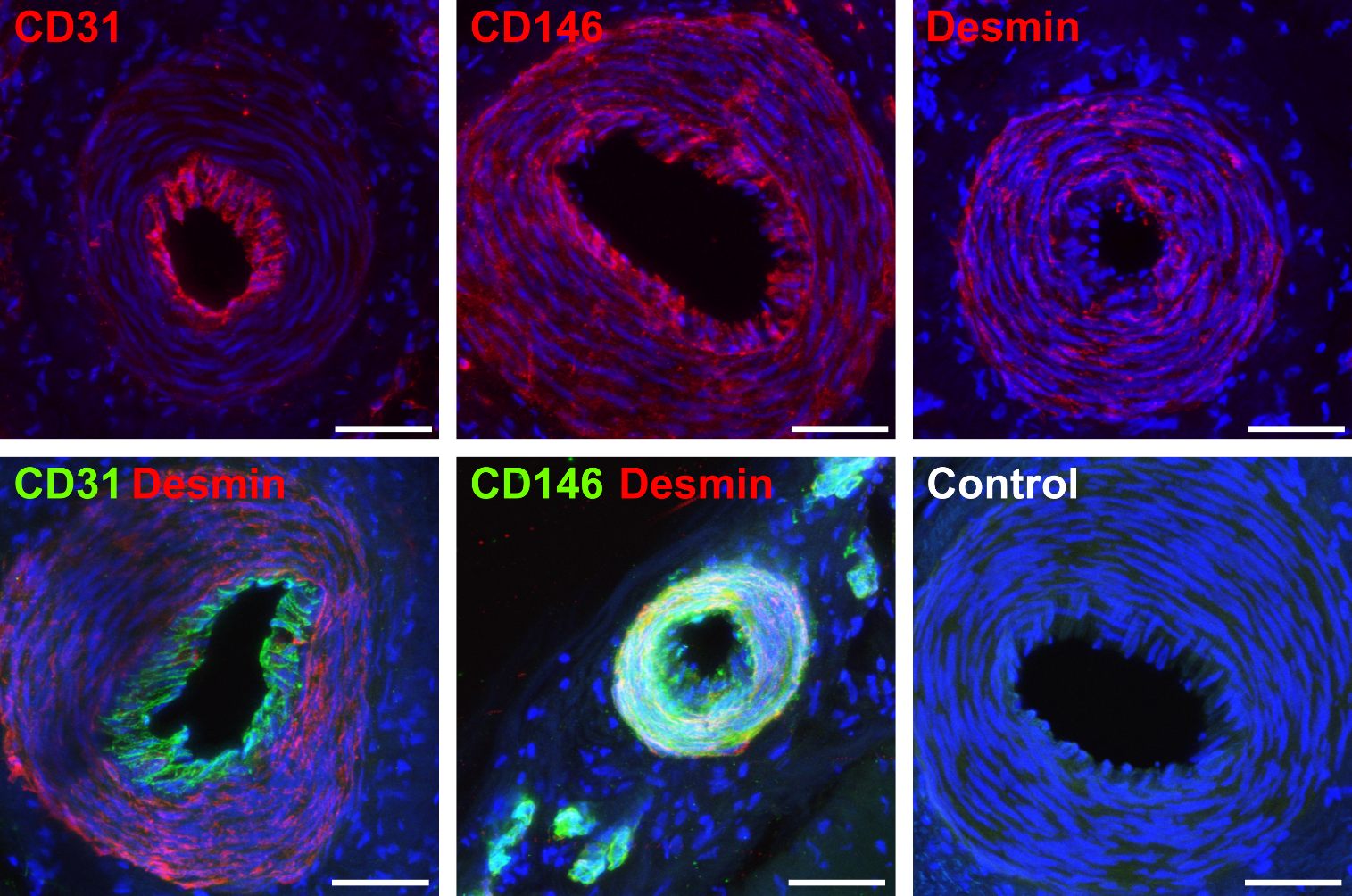


**Supplementary Figure S5.** Epitenon vasculature labelled with endothelial marker CD31, CD146 and pericyte marker Desmin. The reactivity of equine Desmin has been reported previously (Maile et al., 2017). Images represented as maximum projections of 30 µm tissue sections. Nuclei = DAPI (blue). Scale bar = 50 µm.

## Supplementary Tables

**Table S1**. Antibodies used for immunolabelling and their blocking conditions.

| Primary antibody | Supplier | Dilution | Secondary antibody (if applicable) | Supplier (if applicable) | Dilution (if applicable) | Blocking conditions |
| --- | --- | --- | --- | --- | --- | --- |
| CD31 | Abcam (ab28364) | 1:50 | Goat anti-mouse Alexa Fluor® 488 | Thermo Fisher Scientific (A-11001) | 1:500 | 5% goat & 5% horse serum; 1% BSA |
| CD44 | Bio-Rad (MCA2219GA) | 1:100 | Goat anti-mouse Alexa Fluor® 633 | Thermo Fisher Scientific (A-21052) | 1:500 | 5% goat & 5% horse serum; 1% BSA |
| CD90 (THY-1) | Fisher Scientific (15227117) | 1:100 | Goat anti-mouse Alexa Fluor® 633 | Thermo Fisher Scientific (A-21052) | 1:500 | 5% goat & 5% horse serum; 1% BSA |
| CD133 (PROM1) | St. John’s Laboratories (STJ96557) | 1:150 | EnVision peroxidase labelled polymer | Dako (K4065) | N/A  (Used neat) | 5% goat & 5% horse serum; 1% BSA |
| CD146 (MCAM) | Abcam (ab75769) | 1:100 | Goat anti-rabbit Alexa Fluor® 488; Goat anti-rabbit Alexa Fluor® 555; Goat anti-rabbit Alexa Fluor® 594 | Thermo Fisher Scientific (A-11008); Abcam (ab150078); Thermo Fisher Scientific (A-11037) | 1:500 | 5% goat & 5% horse serum; 1% BSA |
| DAG1 | Millipore (05-593) | 1:200 | EnVision peroxidase labelled polymer | Dako (K4065) | N/A  (Used neat) | 5% goat & 5% horse serum; 1% BSA |
| Desmin | Dako (M0760) | 1:50 | Goat anti-mouse Alexa Fluor® 594 | Thermo Fisher Scientific (A-11032) | 1:500 | 5% goat & 5% horse serum; 1% BSA |
| EMCN | St John’s Laboratory (STJ92922) | 1:150 | EnVision peroxidase labelled polymer | Dako (K4065) | N/A  (Used neat) | 5% goat & 5% horse serum; 1% BSA |
| ITGB1 | St. John’s Laboratories (STJ93731) | 1:150 | EnVision peroxidase labelled polymer | Dako (K4065) | N/A  (Used neat) | 5% goat & 5% horse serum; 1% BSA |
| Laminin a4 (LAMA4) | St. John’s Laboratories (STJ93891) | 1:100 | Goat anti-rabbit Alexa Fluor® 594 | Thermo Fisher Scientific (A-11037) | 1:500 | 5% goat & 5% horse serum; 1% BSA |
| Laminin a5 (LAMA5) | St. John’s Laboratories (STJ93892) | 1:150 | EnVision peroxidase labelled polymer | Dako (K4065) | N/A  (Used neat) | 5% goat & 5% horse serum; 1% BSA |
| Mohawk homeobox (MKX) | Insight Bio (ARP32574_P050) | 1:100 | Goat anti-rabbit Alexa Fluor® 555 | Abcam (ab150078) | 1:500 | 5% goat & 5% horse serum; 1% BSA |
| Netrin-1 (NTN1) | St John’s Laboratory (STJ94407) | 1:150 | EnVision peroxidase labelled polymer | Dako (K4065) | N/A  (Used neat) | 5% goat & 5% horse serum; 1% BSA |
| Neuropilin-1 (NRP1) | St John’s Laboratory (STJ94438) | 1:150 | EnVision peroxidase labelled polymer | Dako (K4065) | N/A  (Used neat) | 5% goat & 5% horse serum; 1% BSA |
| Pan-laminin | Abcam (ab11575) | 1:500 | EnVision peroxidase labelled polymer | Dako (K4065) | N/A  (Used neat) | 5% goat & 5% horse serum; 1% BSA |
| Perlecan | Fisher Scientific (MA514641) | 1:500 | EnVision peroxidase labelled polymer | Dako (K4065) | N/A  (Used neat) | 5% goat & 5% horse serum; 1% BSA |
| Type IV Collagen | Southern Biotech (1340-01) | 1:200 | Rabbit anti-goat immunoglobulins/HRP | Dako (P0160) | 1:200 | 5% rabbit & 5% horse serum; 1% BSA |
| VWF | Dako (A0082) | 1:150 | EnVision peroxidase labelled polymer | Dako (K4065) | N/A  (Used neat) | 5% goat & 5% horse serum; 1% BSA |

# References

Maile, C.A., Hingst, J.R., Mahalingan, K.K., O'Reilly, A.O., Cleasby, M.E., Mickelson, J.R., et al. (2017). A highly prevalent equine glycogen storage disease is explained by constitutive activation of a mutant glycogen synthase. *Biochimica et Biophysica Acta (BBA) - General Subjects* 1861(1)**,** 3388-3398. doi: 10.1016/j.bbagen.2016.08.021.
